# Blue light‐induced stomatal opening is associated with species‐specific changes in primary metabolism but not with starch breakdown in guard cells

**DOI:** 10.1111/nph.70257

**Published:** 2025-06-11

**Authors:** Humaira Bahadar, Eva Gomes Morais, Francisco Bruno S. Freire, Valéria F. Lima, Marina Ellen Giacomelli, Leticia dos Anjos, Werner Camargos Antunes, Danilo M. Daloso

**Affiliations:** ^1^ Department of Biochemistry and Molecular Biology Federal University of Ceará Fortaleza 60451‐970 Brazil; ^2^ Department of Biology, Plant Ecophysiological Laboratory State University of Maringá Maringá 87020‐900 Brazil

**Keywords:** *Arabidopsis thaliana*, metabolic regulation, *Nicotiana tabacum*, stomatal movement regulation, *Vigna unguiculata*

## Abstract

- Blue light (BL)‐induced stomatal opening has been associated with starch breakdown within Arabidopsis guard cells (GCs). However, whether this mechanism is conserved in angiosperms and which metabolic pathways are activated downstream of BL perception and/or starch degradation, remains unknown.
- Here, we performed stomatal and metabolomics analyses to investigate how BL stomatal responses are associated with GC starch and primary metabolisms in Arabidopsis, cowpea, and tobacco.
- The stomatal aperture increased, but no starch degradation was observed in all species under BL. Guard cell primary metabolism was altered by BL exposition in a species‐specific and time‐dependent manner. Sucrose was negatively correlated with stomatal aperture in both Arabidopsis and cowpea, resembling previous results during white light (WL)‐induced stomatal opening. However, contrasting metabolic changes were observed in tobacco under BL and WL. For instance, malate and fumarate accumulated preferentially in tobacco GCs under BL and WL, respectively. Despite the species‐specific BL metabolic responses, sugars were positively correlated with tricarboxylic acid cycle‐related metabolites in all species under BL, similar to those previously observed under WL‐induced stomatal opening.
- Our study highlights that both starch breakdown and the changes in primary metabolism within GCs triggered by light depend on the species, environmental condition, and/or light quality.

## Abstract 2

See also the Commentary on this article by Fernie & Timm, **248**: 2184–2186.

## Introduction

Plants have many microscopic adjustable pores, known as stomata, which are surrounded by two guard cells (GCs) on their aerial surface. Changes in GC metabolism regulate the opening or closure of stomatal pores in response to endogenous and environmental cues (Sussmilch *et al*., 2019). Stomatal opening allows the influx of CO_2_ for photosynthesis and the efflux of water through transpiration. Thus, while stomatal opening is important to optimize photosynthesis and consequently plant growth, stomatal closure is key to avoid dehydration during drought periods (Lima *et al*., 2018). Understanding the mechanisms that regulate the interplay between photosynthesis and stomatal movements has direct implications for plant metabolic engineering, especially in the current climate change scenario (Evans & Lawson, 2020). However, the regulation of stomatal movements is highly complex, involving autonomous responses of GCs such as direct perception of light and CO_2_ as well as responses triggered by stimuli such as phytohormones and metabolites from other cell types, especially subsidiary (whenever present) and mesophyll cells (Flütsch & Santelia, 2021).

Stomatal opening is induced by light, with specific mechanisms and signalling pathways that regulate responses to red light (RL) and blue light (BL) (Shimazaki *et al*., 2007). Initial studies suggested RL's role might be associated with signals derived from mesophyll photosynthetic activity (Mott *et al*., 2008; Mott, 2009). Indeed, recent results showed that RL directly induces the phosphorylation of GC plasma membrane H^+^‐ATPases in a photosynthesis‐dependent manner (Ando & Kinoshita, 2018). Similarly, BL‐induced stomatal opening involves H^+^‐ATPases activation in GC plasma membrane, in a process dependent on the phototropin‐mediated BL perception. This activation establishes a H^+^ gradient across GC plasma membrane, facilitating the influx of K^+^ and anions into the guard cells (Ding *et al*., 2021). Simultaneously, metabolic changes occur within GCs to produce osmolytes and the energy needed for: metabolism; H^+^‐ATPase activation; and several ion channels present at GC tonoplast and plasma membranes (Daloso *et al*., 2017). It is expected therefore that substantial changes occur in GC metabolism after BL perception. Evidence suggests that both starch and lipids are degraded within Arabidopsis GCs upon BL exposition (Horrer *et al*., 2016; McLachlan *et al*., 2016). However, which metabolic pathways are activated downstream of starch and lipid degradation remains unknown.

Among the metabolic pathways involved in stomatal movement regulation, starch, sugars, and organic acids have historically received special attention (Daloso *et al*., 2017; Dang *et al*., 2024). Initially, Hugo von Mohl suggested that the accumulation of osmolytes within GCs modulates the turgor changes in these cells and consequently stomatal movements (Von Mohl, 1856). Later, Francis Ernst Lloyd proposed that the interplay between the synthesis of starch and sugars would be a mechanism to induce stomatal closure and opening, respectively (Lloyd, 1908), giving rise to the starch–sugar theory. However, after the discoveries that potassium (K^+^) is a key osmolyte and malate may act as a counter ion of K^+^ for GC osmoregulation (Fischer, 1968; Allaway, 1973; Outlaw & Lowry, 1977), several studies proposed that starch could be the source for malate synthesis in GCs during stomatal opening (Ogawa *et al*., 1978; Outlaw & Manchester, 1979; Schnabl, 1980; Lasceve *et al*., 1997). On the other hand, the activation of gluconeogenesis and starch synthesis would occur during stomatal closure conditions (Dittrich & Raschke, 1977; Van Kirk & Raschke, 1978; Schnabl, 1980; Penfield *et al*., 2012). These studies collectively indicate that the dynamic of GC carbohydrate and organic acid metabolisms is pivotal for stomatal movement regulation, but the detailed mechanisms by which starch synthesis and degradation underpin stomatal movement regulation remain unclear.

The importance of GC starch degradation for stomatal opening is further supported by findings showing that: (1) starch content was quantitatively related to stomatal aperture in *Vicia faba* (Outlaw & Manchester, 1979); (2) BL‐induced stomatal opening was disrupted in starch‐deficient guard cells in an Arabidopsis mutant (Lasceve *et al*., 1997); and (3) the Arabidopsis double mutant *amy3 bam1* (α‐amylase 3, β‐amylase 1) is impaired in both starch degradation within GCs and in BL‐induced stomatal opening (Horrer *et al*., 2016; Flütsch *et al*., 2020). These studies suggest that starch mobilization upon BL perception plays an important role for a rapid and efficient stomatal opening. However, several other studies showed that GC starch content was not correlated with stomatal aperture under white light (WL) or BL (Heath, 1947; Ogawa, 1981; Tallman & Zeiger, 1988; Daloso *et al*., 2015). Furthermore, no study to date has performed a detailed metabolic characterization of GCs during BL‐induced stomatal opening. Here, we investigated the effects of BL exposure on stomatal movements and in GC starch and primary metabolisms in cowpea and tobacco plants.

## Materials and Methods

### Plant material and growth conditions

Cowpea (*Vigna unguiculata* L. Walp.) seeds were surface‐sterilized with 70% ethanol followed by washing with distilled water and sown on a germination paper, with 10 seeds per sheet. The seeds were kept inside a plastic bag for 5 d, then the seedlings were transplanted into pots (4‐l) containing vermiculite and sand (1 : 1) and maintained in a glasshouse. Arabidopsis (*Arabidopsis thaliana* L.) and tobacco (*Nicotiana tabacum* L.) seeds were surface‐sterilized with 70% ethanol followed by washing with distilled water. Tobacco seeds were sown on a substrate composed of sand, vermiculite, and soil (4 : 2 : 1) in a seed germination tray. After 1 wk, tobacco plants were transferred to 4‐l pots and kept in a glasshouse. Arabidopsis were sown in 0.1‐l pots containing a commercial organic substrate (Topstrato®) and maintained in a growth room under short‐day conditions (8 h : 16 h, light : dark), daily average temperature 20–22°C, and artificial WL (160 μmol photons m^−2^ s^−1^). Cowpea and tobacco plants were irrigated with Hoagland's solution (Hoagland & Arnon, 1950) twice per week, while Arabidopsis was irrigated with this solution once per week. Fully expanded cowpea and tobacco leaves and whole Arabidopsis rosettes were harvested predawn for GC‐enriched epidermal fragments isolation.

### Guard cell‐enriched epidermal fragments isolation

A pool of GC‐enriched epidermal fragments (here referred to solely as GCs) was obtained according to a protocol optimized for metabolomics analysis (Daloso *et al*., 2015). Guard cell isolation was carried out using three cowpea and tobacco leaves and two Arabidopsis rosettes per replicate. Both primary and secondary veins were removed from tobacco leaves, while only the main nervure was removed from cowpea leaves. The leaves and Arabidopsis rosettes were subjected to three pulses (1 min each) of blending in a blender (Philips, RI, 2044 B.V.; International Philips, Amsterdam, the Netherlands) equipped with an internal filter to facilitate the separation of GCs from fibres, mesophyll cells, and other cell debris. After blending, GCs were collected in a nylon membrane (200 μm) and washed with distilled water to remove contaminants. Subsequently, GCs were transferred to a hypertonic solution (0.5 M mannitol) under dark conditions to prevent stomatal opening. These steps were repeated until a sufficient pool of GCs was obtained for subsequent stomatal aperture and metabolomics analyses. Another set of GCs was harvested predawn and not transferred to the hypertonic solution, being immediately transferred to BL. After 0, 10, 20, 30, 40, and/or 60 min under BL for cowpea and tobacco and 0 and 60 min for Arabidopsis, GCs were collected in a nylon membrane. A set of GCs was used to measure stomatal aperture and another set frozen in liquid nitrogen for starch and metabolomics analyses.

### Stomatal aperture kinetics under blue light

Guard cells harvested predawn were collected from the hypertonic solution, extensively washed with distilled water, transferred to Petri dishes containing a stomatal opening solution (5 mM KCl + 5 mM NaOH + 50 μM CaCl_2_, pH 6.5), and then exposed to BL (75–90 μmol photons m^−2^ s^−1^). After 0, 10, 20, 30, 40, and 60 min under BL, GCs were collected on a nylon membrane, rapidly washed to remove excess buffer, and then frozen in liquid nitrogen. The samples were stored at −80°C until further analysis. Stomatal aperture was determined using a light microscope with a coupled digital camera. At least 40–60 stomata were measured for each replicate. The width and length of the stomatal pore were measured using the ImageJ software (http://fiji.sc/), as described earlier (Medeiros *et al*., 2018).

### Stomatal conductance kinetics under blue light

Cowpea and tobacco plants were grown under controlled conditions (25°C, 12 h photoperiod, and 200 μmol photons m^−2^ s^−1^ of white light) for 15 d. The stomatal conductance (*g*_s_) kinetics were carried out in dark‐adapted plants and at the morning period of the day. The *g*_s_ was measured in the most expanded leaf using a portable infra‐red gas analyser (IRGA) (LiCor 6800, Lincoln, NE, USA) containing a clear‐top 9 cm^2^ leaf chamber (Clear‐top Leaf Chamber, Li6800‐12A). The *g*_s_ was measured for 10 min in the dark, followed by 90 min under 100 μmol m^−2^ s^−1^ of BL (450 nm) and further 30 min in the dark. The BL was supplemented by a LED platform placed above the IRGA leaf chamber. Detailed spectral analysis of the light sources used here were described previously (Falcioni *et al*., 2020; Pattaro *et al*., 2024). The temperature, relative humidity, and CO_2_ concentration in the IRGA leaf chamber were 25°C, 60%, and 400 parts per million, respectively. The speed of stomatal opening in response to BL was estimated as the slope of the linear phase of the stomatal opening kinetics, as described earlier (Lima *et al*., 2019).

### Gas chromatography mass spectrometry‐based metabolite profiling analysis

Approximately 500 mg of powdered GC‐enriched epidermal fragments was used for metabolite extraction. The extraction and derivatization of polar metabolites were carried out using a well‐established protocol (Lisec *et al*., 2006), with slight modifications (Lima *et al*., 2021). The metabolites were analysed by gas chromatography coupled to mass spectrometry (GC‐MS; QP‐PLUS 2010, Shimadzu, Japan), as described earlier (Lisec *et al*., 2006). The mass spectra obtained were analysed using Xcalibur® 2.1 (Thermo Fisher Scientific, Waltham, MA, USA). Metabolites were identified using the Golm Metabolome Database (http://gmd.mpimp‐golm.mpg.de/) (Kopka *et al*., 2005). Metabolite levels were normalized by the level of ribitol and fresh weight (FW) used for extraction. These data can be found in Supporting Information Dataset S1.

### Starch quantification

Starch levels were measured using a spectrophotometer as described previously (Trethewey *et al*., 1998). Starch was quantified in the pellet remaining after metabolite extraction for metabolite profiling. The pellet was washed three times with 80% ethanol at 70°C until a clear pellet was obtained. Starch in the pellet was then digested by the addition of 400 μl of 0.2 M KOH at 90°C for 1 h and neutralized with 210 μl of 1 M acetic acid. Subsequently, 100 μl of this solution was used for starch digestion using citrate buffer (0.3 M) at pH 5.0 and the enzymes amyloglucosidase (1 U reaction^−1^) and α‐amylase (0.1 U reaction^−1^) at 55°C overnight in a final volume of 580 μl. Starch was quantified using an enzymatic method coupled to NADH production measured at 340 nm using a commercially available kit (Glucose Assay Kit; Sigma‐Aldrich). Starch levels were quantified based on a standard curve of glucose.

### Statistical analyses

Data are presented as the mean of four replicates ± SD. Significant differences between different time points were determined by one‐way ANOVA and Tukey's test (*P* < 0.05). Punctual comparisons were carried out by Student's *t*‐test (*P* < 0.05). Metabolomics data were analysed using the MetaboAnalyst platform (Pang *et al*., 2021). Pearson's correlation analysis and partial least‐squares discriminant analysis (PLS‐DA) were performed on cube‐root‐transformed and mean‐centred data (for cowpea data), log transformation and auto‐scaling mode (for tobacco data), and cube‐root‐transformed and auto‐scaling mode (for Arabidopsis data) (Xia & Wishart, 2011). Heat maps were created using the MeV 4.9.0 software. K‐means clustering based on Euclidian distance was carried out using stomatal aperture and metabolite profiling data. The number of clusters was manually determined according to the dynamic of accumulation/degradation of the metabolites in both species. This analysis was carried out using the MetaboAnalyst platform.

## Results

### Blue light‐induced stomatal opening was not associated with changes in starch content in Arabidopsis, cowpea, and tobacco guard cells under our experimental conditions

**Fig. 1:**
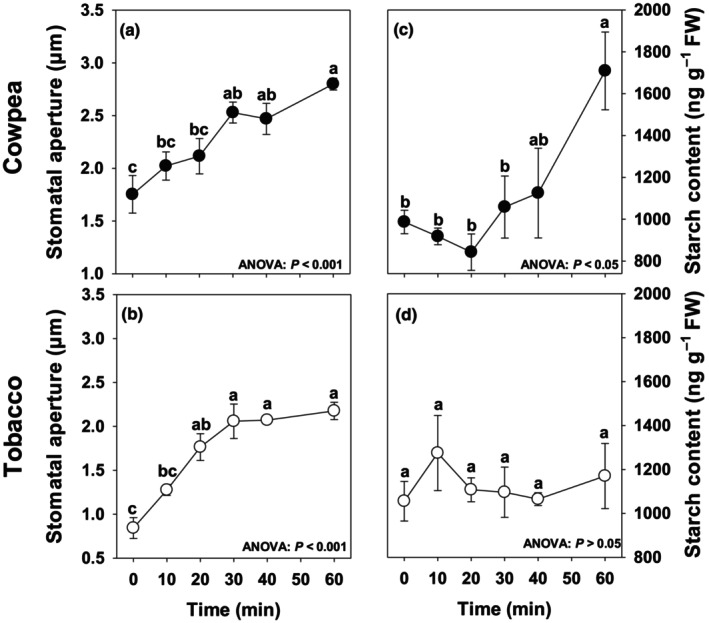
Stomatal aperture (a, b) and starch content (c, d) in guard cells (GCs) of cowpea (a, c) and tobacco (b, d) throughout time under blue light (BL). A pool of GC‐enriched epidermal fragments was harvested predawn, stored in a hypertonic solution to avoid stomatal opening, and then transferred to BL (75–90 μmol photons m^−2^ s^−1^). After 0, 10, 20, 30, 40, and 60 min, GCs were harvested in a membrane and rapidly washed with distilled water. One set of GCs was immediately used to measure stomatal aperture using a light microscope, and another set was rapidly frozen in liquid nitrogen for starch analysis. Stomatal aperture data refer to the width of the stomatal pore (μm). Guard cell starch content (ng g^−1^ FW) was determined spectrophotometrically. Significant differences throughout time are indicated by different letters, as determined by ANOVA and Tukey's test (*P* < 0.05). Data are mean ± SE (*n* = 4).

Stomatal aperture increased significantly from 0 to 60 min in Arabidopsis (Fig. S1a). In cowpea and tobacco, stomatal aperture increased linearly from 0 to 30 min, when then a plateau was observed between 30 and 60 min (Fig. 1a,b). We next investigated stomatal responses to BL by gas exchange analysis. The kinetics of stomatal conductance (*g*_s_) was analysed by an infra‐red gas analyser in plants under BL (100 μmol photons m^−2^ s^−1^). The results further demonstrated that the stomata from tobacco and cowpea are responsive to BL, but both the magnitude and the speed of the increases in *g*_s_ were higher in tobacco than cowpea (Fig. S2a,b).

Starch content did not change either in Arabidopsis or in tobacco GCs, while cowpea GCs showed higher content of starch after 60 min (Figs S21b, 1c,d). The lack of starch degradation in the first 60 min under BL does not corroborate previous findings in Arabidopsis GCs, in which a rapid decrease in starch level (measured as starch granule area from microscopic images) was observed in the first 60 min under BL (Horrer *et al*., 2016; Flütsch *et al*., 2020). This discrepancy could be associated with: (1) the kinetics of starch degradation, which frequently requires several minutes to hours; (2) the plant growth condition; (3) the differences in the methods used for starch determination; and/or (4) the experimental procedure used here, in which a pool of GC‐enriched epidermal fragments was obtained and stored in a hypertonic solution before the stomatal kinetic experiment. This last procedure is necessary to collect sufficient GCs for metabolomics analysis (Daloso *et al*., 2015).

We then carried out other experiments to address some of these possibilities. First, dark‐adapted cowpea and tobacco plants were subjected to BL, and GCs were isolated and immediately frozen after 0 and 60 min under BL, eliminating the step of pooling GCs in a hypertonic solution. The results were consistent with those in the previous experiment, with no difference in starch content between GCs harvested predawn (0 min) and after 60 min under BL, although a tendency to decrease starch content after 60 min of BL exposition was observed in both species, especially in tobacco (*P* = 0.05) (Fig. S3a,b). In Arabidopsis, we determined GC starch content after 0 and 60 min under BL in predawn‐harvested guard cells that were not submitted to the hypertonic solution. No starch degradation was observed in Arabidopsis GCs after 60 min under BL (Fig. S3c), indicating that either our experimental conditions or our method to quantify starch lead to discrepant results from previous studies (Horrer *et al*., 2016; Flütsch *et al*., 2020). Taken together, our stomatal aperture and starch results suggest that BL‐induced stomatal opening does not involve starch remobilization in Arabidopsis, cowpea, and tobacco GCs, but the level of starch after 60 min under BL ranges according to the experimental condition.

### Guard cell metabolic changes triggered by blue light exposition

We next carried out a metabolite profiling analysis to unravel the BL‐induced changes in Arabidopsis, cowpea, and tobacco GC primary metabolism using a well‐established gas chromatography mass spectrometry approach (Lisec *et al*., 2006). Guard cells were extracted at predawn, pooled in a hypertonic solution, washed extensively, transferred to BL, and harvested after 0 and 60 min for Arabidopsis and 0, 10, 20, 30, 40, and 60 min for both cowpea and tobacco. In Arabidopsis GCs, the level of 15 out of the 17 metabolites identified decreased after 60 min under BL, when compared to samples frozen at 0 min (Fig. S4a). PLS‐DA demonstrated that BL substantially alters Arabidopsis GC metabolism, as evidenced by the clear separation of 0‐ and 60‐min samples by the PC1 (Fig. S4b).

**Fig. 2:**
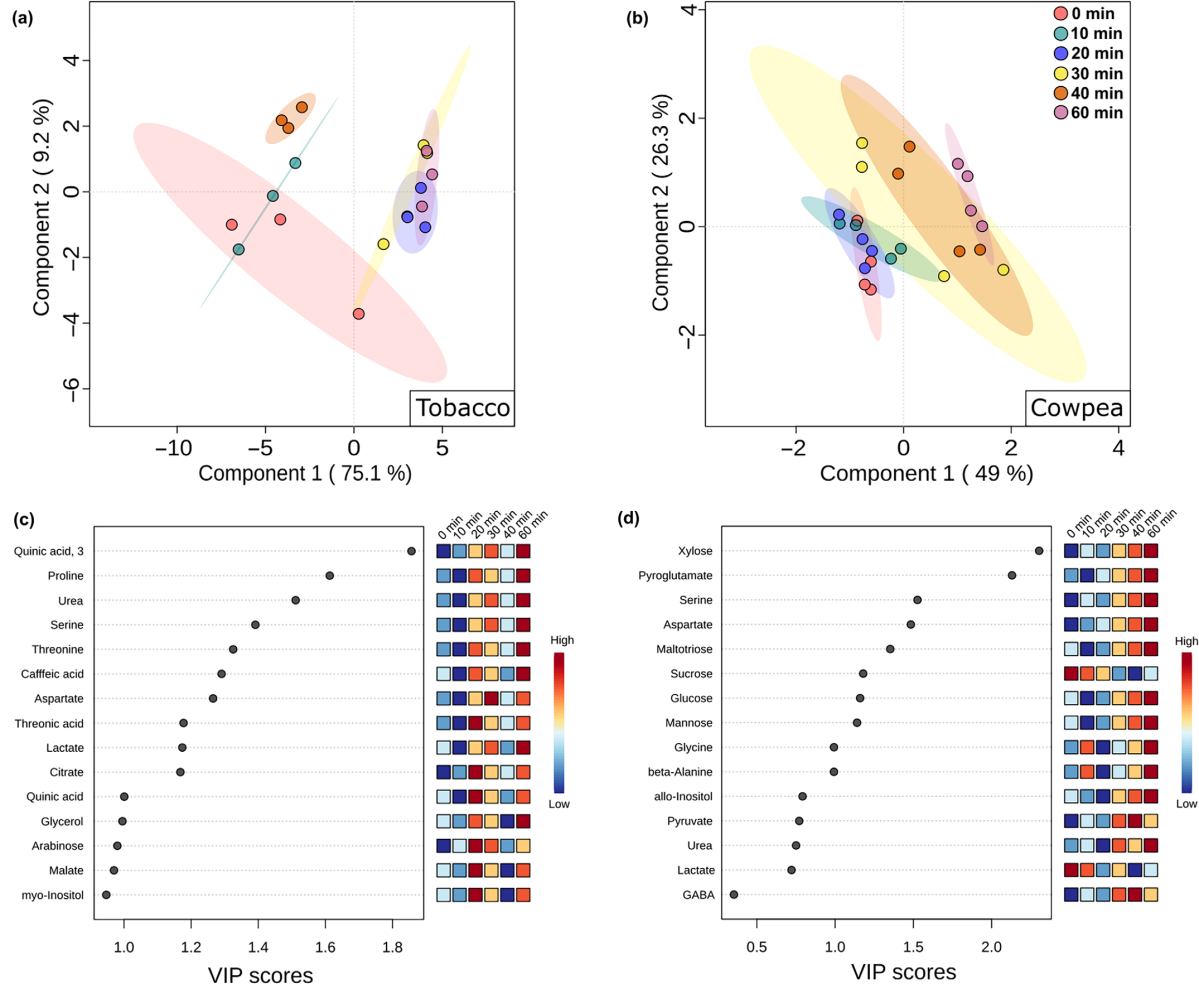
Changes in guard cell (GC) primary metabolites of cowpea and tobacco induced by blue light (BL). (a, b) Partial least‐squares discriminant analysis (PLS‐DA) using metabolite profiling data from tobacco (a) and cowpea (b) GCs harvested after 0, 10, 20, 30, 40, and 60 min under BL. The percentage variation explained by the Components 1 and 2 of PLS‐DAs are represented in each axis. (c, d) Variable importance in projection (VIP) scores from PLS‐DA using metabolite profiling data from tobacco (c) and cowpea (d) GCs. The metabolites of the VIP score list are ranked from top to down as the most important for each PLS‐DA model. These analyses were carried out using the Metaboanalyst platform (*n* = 3–4).

Only three metabolites (glycerol, threonine, and 3‐caffeoylquinic acid) increased over time in tobacco GCs (Fig. S5a). By contrast, six metabolites (glycine, β‐alanine, serine, aspartate, pyroglutamate, and maltotriose) increased over time in cowpea GCs, while lactate showed a lower level at time 20 min than at time 0 min (Fig. S5b). Although these results suggest minor BL‐induced changes in GC metabolite pool, especially in tobacco, PLS‐DA separated the time points under BL from the time 0 min. The time points 40 and 60 min were clearly separated from the time 0 min by the first component in cowpea, while these time points were separated by the second and first components in tobacco, respectively (Fig. 2a,b). Notably, the separation among the time points was clearly time‐dependent in cowpea, with the last time points (40 and 60 min) more separated from the time 0 min (Fig. 2b). By contrast, tobacco GCs showed three distinct clusters (Clt) composed of the times 0 min (Clt1), 40 min (Clt2) and 20, 30, and 60 min (Clt3), with time 10 min positioned between 0 and 40 min (Fig. 2a). The variable importance in projection (VIP) list highlights the metabolites that mostly contributed to the observed separation in PLS‐DA, in which metabolites with VIP score higher than 1 are considered good representatives (Xia & Wishart, 2011). While cowpea metabolites with a VIP score higher than 1 showed a pattern of continuous increase or decrease over time, tobacco metabolites showed a highly heterogeneous dynamic over time. For instance, the level of all metabolites in the VIP score list increased from 0 to 30 min, decreased from 30 to 40 min, and increased again from 40 to 60 min in tobacco (Fig. 2c,d).

### Metabolites associated with the dynamic of blue light‐induced stomatal opening

**Fig. 3:**
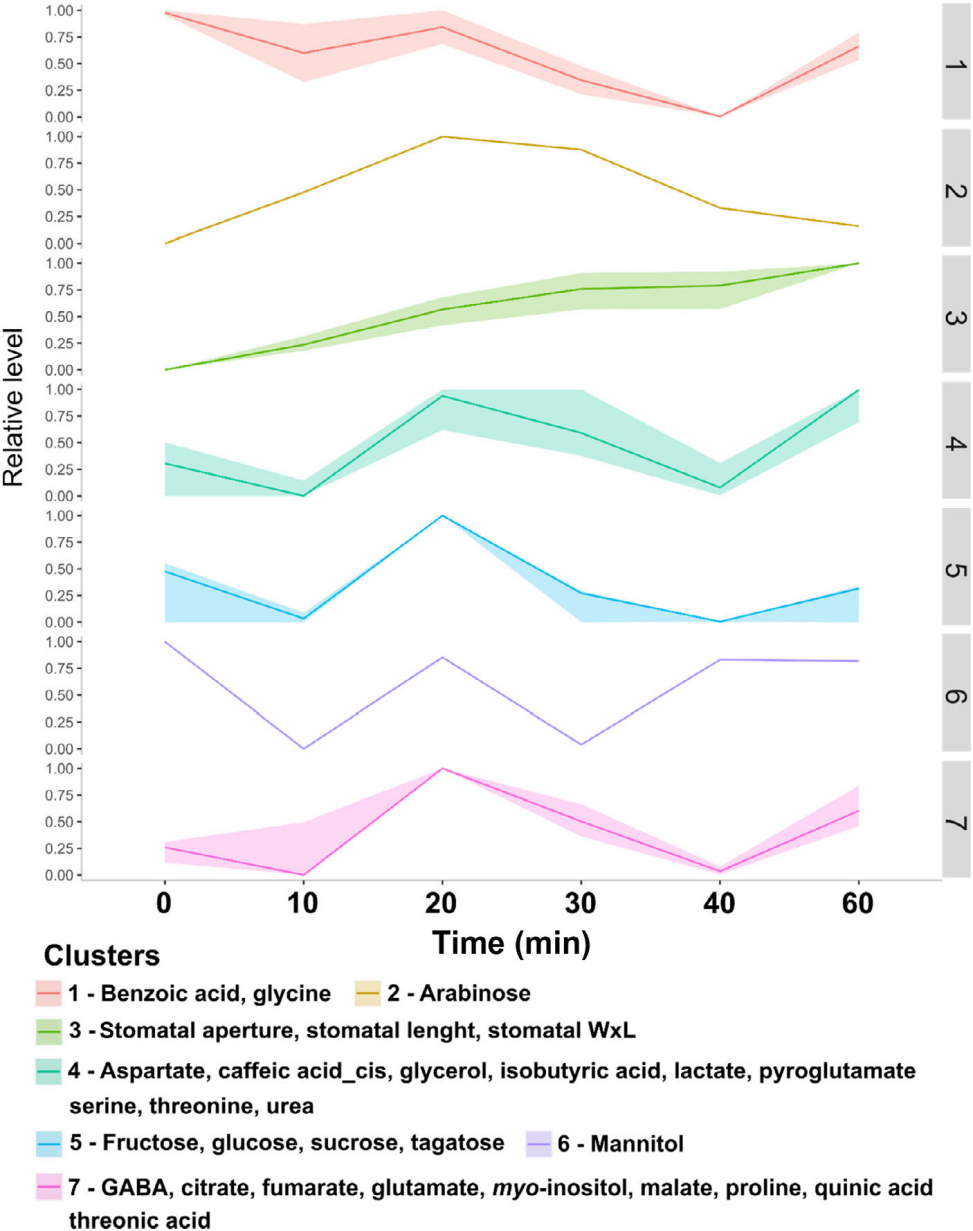
K‐means clustering analysis using tobacco stomatal aperture and metabolite profiling data normalized using a minimum (0)–maximum (1) transformation. Seven (1–7) clusters were generated, which combine parameters with a similar increasing or decreasing trend throughout the time of the experiment (i.e. from 0 to 60 min). The *Y*‐axis highlights the relative level of the parameters, in which the minimum and maximum values observed between 0 and 60 min were set to 0 or 1, and the values in between were then proportionally normalized between 0 and 1 throughout time. This analysis was carried out using the Metaboanalyst platform (*n* = 4). stomatal WxL, stomatal width × length.

**Fig. 4:**
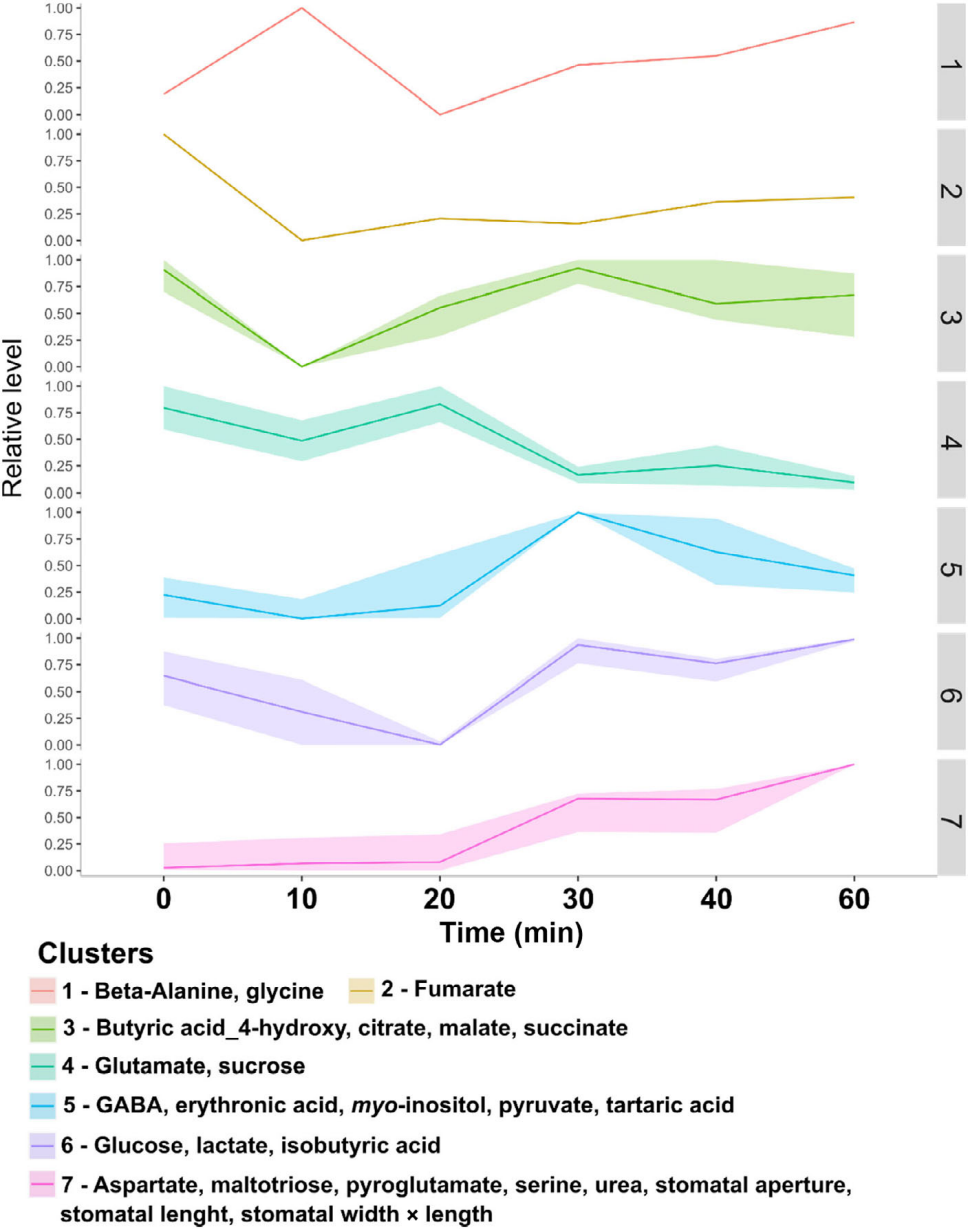
K‐means clustering analysis using cowpea stomatal aperture and metabolite profiling data normalized using a minimum (0)–maximum (1) transformation. Seven (1–7) clusters were generated, which combine parameters with a similar increasing or decreasing trend throughout the time of the experiment (i.e. from 0 to 60 min). The *Y*‐axis highlights the relative level of the parameters, in which the minimum and maximum values observed between 0 and 60 min were set to 0 or 1, and the values in between were then proportionally normalized between 0 and 1 throughout time. This analysis was carried out using the Metaboanalyst platform (*n* = 4).

In order to investigate which metabolites are associated with the dynamic of BL‐induced stomatal opening, we employed a maximum–minimum transformation of both stomatal aperture and metabolite profiling data (Cândido‐Sobrinho *et al*., 2022) and carried out a K‐means clustering analysis, which combines parameters with similar patterns of increase/decrease throughout time (Szecowka *et al*., 2013). Seven clusters (Clt1‐7) were generated for each species. The composition and the dynamics of metabolite accumulation/degradation diverged substantially between tobacco and cowpea (Figs 3, 4). In tobacco, stomatal aperture parameters (Clt3) were not clustered with any metabolite, and none of the other clusters had a clear opposite dynamic than stomatal aperture. The dynamics of Clt1, 2, 4, 5, 6, and 7 exhibited high variability over time (Fig. 3), consistent with previous results from tobacco GCs under WL (Daloso *et al*., 2015). By contrast, stomatal aperture parameters clustered with aspartate, maltotriose, pyroglutamate, serine, and urea in cowpea (Clt7) (Fig. 4). The maximum level of fumarate was observed at time 0 min, decreased at 10 min, and then remained relatively constant until 60 min (Clt2). Interestingly, Clt4 showed an opposite dynamic while Clusters 1, 3, 5, and 6 showed a trend similar to Clt7 from 20 to 60 min (Fig. 4). This analysis indicates that sucrose and glutamate (Clt4) are degraded while glucose, maltotriose, and pyroglutamate are synthesized during the BL‐induced stomatal opening in cowpea.

Further analysis showed negative correlations between sucrose and stomatal aperture parameters, while certain sugars and amino acids were positively correlated in cowpea (Fig. S6). In tobacco, 3‐caffeoylquinic acid, caffeic acid, lactate, urea, proline, and glycine showed positive correlations with stomatal aperture parameters (Fig. S7). In Arabidopsis, stomatal aperture was negatively correlated with the level of citrate, serine acetyl, sucrose, *myo‐*inositol, γ‐Aminobutanoic acid, benzoic acid, erythronic acid, and fumarate (Fig. S8). Sucrose and glucose were positively correlated with metabolites of the tricarboxylic acid (TCA) cycle, such as citrate, malate, and fumarate in cowpea and tobacco. In Arabidopsis, fumarate was positively correlated with fructose, glucose, and sucrose, and fructose was further positively correlated with malate (Figs S6–S8). These results suggest that the dynamic of the BL‐induced stomatal opening is associated with different metabolic changes in Arabidopsis, tobacco, and cowpea.

### Blue light‐induced changes in guard cell primary metabolism are species‐specific and time‐dependent

**Fig. 5:**
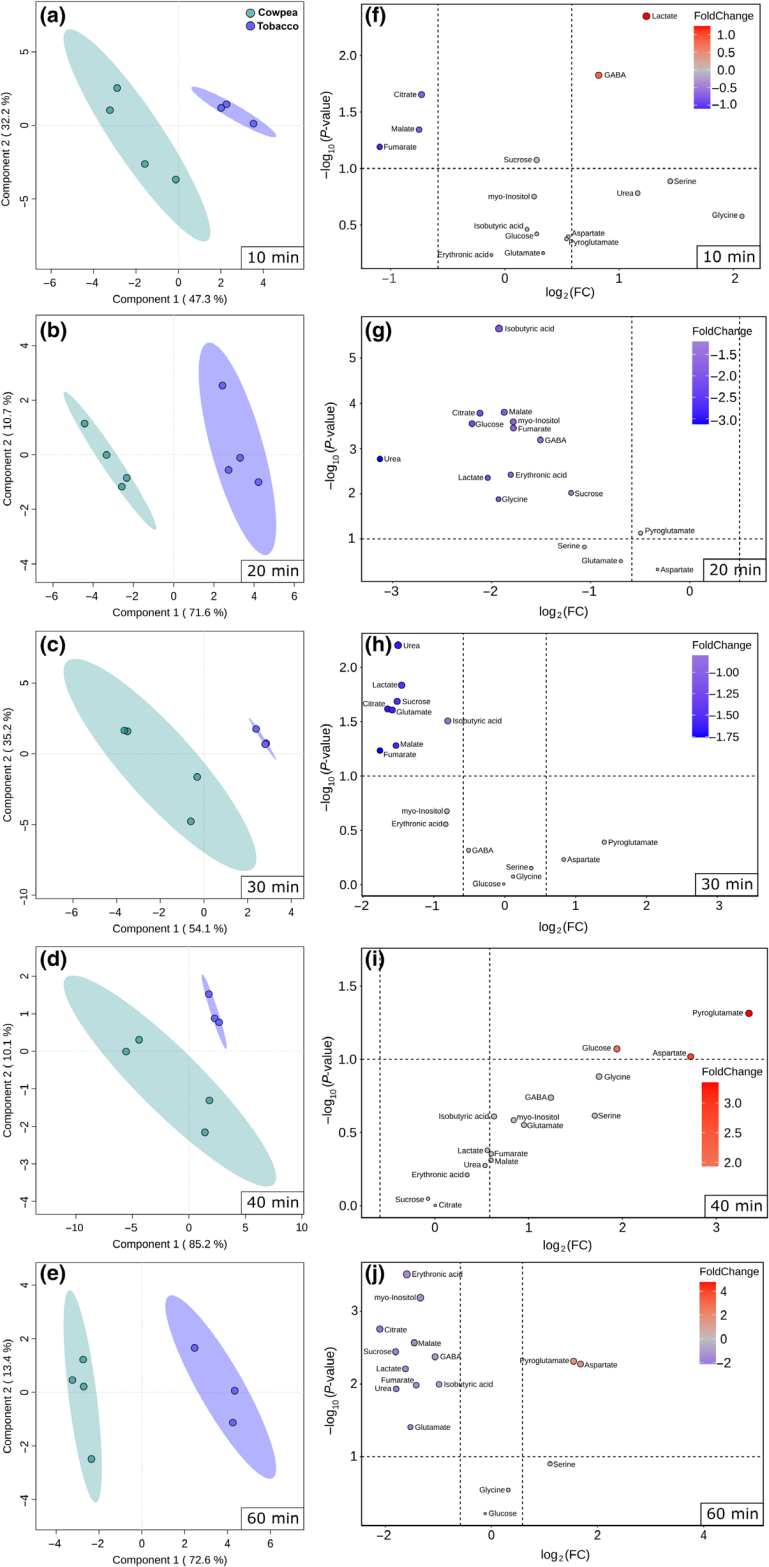
Partial least‐squares discriminant analysis (a–e) and volcano plots (f–j) comparing the relative metabolic changes between tobacco and cowpea guard cells subjected to 0, 10, 20, 30, 40, and 60 min of blue light. These analyses were carried out using the metabolite profiling data normalized according to the time 0 min within each genotype. (a–e) The percentage variation explained by Components 1 and 2 are represented in each axis. (f–j) Metabolites in blue and red colour have lower and higher level in cowpea than in tobacco, respectively, by Student's *t*‐test (*P* < 0.05). These analyses were carried out using the Metaboanalyst platform (*n* = 4).

**Fig. 6:**
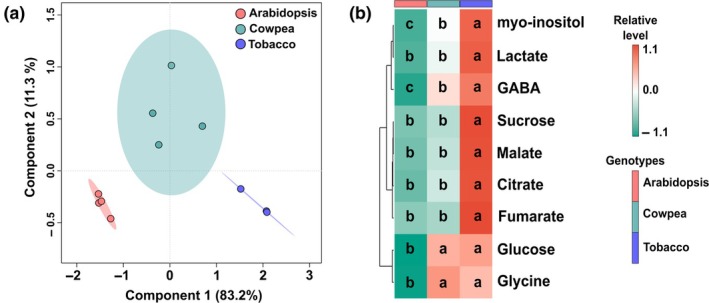
Comparison of the guard cell (GC) metabolic changes triggered by blue light (BL) among Arabidopsis, cowpea, and tobacco. This comparison was carried out using metabolite profiling data from GCs subjected to 60 min of BL normalized according to the values obtained at the time 0 min of each genotype. These relative data were then used to perform a partial least‐squares discriminant analysis (PLS‐DA) (a) and represented as a heatmap (b). The relative alteration in each metabolite was compared among species by ANOVA and Tukey's test. Different letters in each metabolite row indicate significant difference among the genotypes (*P* < 0.05). The percentage variation explained by the PC1 and the PC2 of PLS‐DAs are represented in each axis. PLS‐DA was carried out using the Metaboanalyst platform (*n* = 4).

We next normalized the metabolite profiling data according to the control (0 min) of each genotype. PLS‐DA using these relative data confirmed substantial differences in BL‐induced metabolic changes between cowpea and tobacco, as evidenced by the clear separation of these species by Component 1 in each time point (Fig. 5a–e). The level of malate, citrate, and fumarate (among other metabolites) was lower, while that of pyroglutamate, glucose, aspartate, GABA, and lactate was higher in cowpea than in tobacco in at least one of the time points analysed here (Fig. 5f–j). We next took nine metabolites commonly detected in Arabidopsis, cowpea, and tobacco and normalized their level found at 60 min with their respective control (0 min). PLS‐DA using these data demonstrated that three different clusters were formed (Fig. 6a). The level of the nine metabolites was higher in tobacco than in Arabidopsis, while cowpea presented an intermediate phenotype among the species (Fig. 6b). These results collectively indicate that BL exposure altered GC primary metabolism in a species‐specific and time‐dependent manner.

### Tobacco guard cell metabolic responses differ between blue and white light

**Fig. 7:**
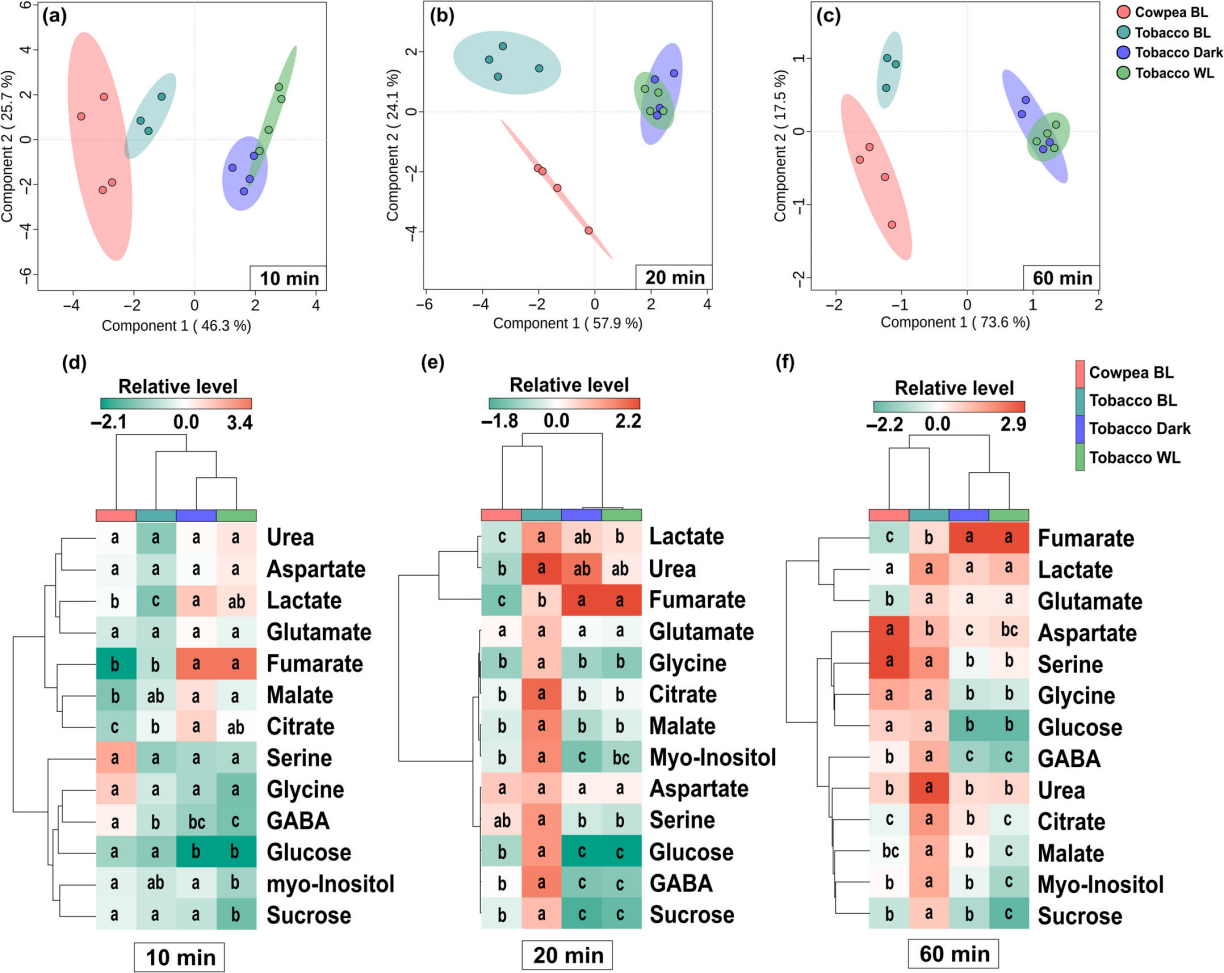
Partial least‐squares discriminant analysis (PLS‐DA) (a–c) and heat map representation (d–f) comparing the guard cell (GC) metabolic changes induced by white light (WL), blue light (BL), and extended darkness (Dark). These analyses were carried out using metabolite profiling data from 10 (a, d), 20 (b, e), and 60 min (c, f) normalized according to the values obtained at time 0 min within each genotype and treatment. Guard cells were collected at predawn, transferred to plates, and maintained under darkness or transferred to WL (400 μmol photons m^−2^ s^−1^) or BL (75–90 μmol photons m^−2^ s^−1^) for 0, 10, 20, and 60 min. The percentage variation explained by the PC1 and the PC2 of PLS‐DAs is represented on each axis. The relative alteration in each metabolite was compared among species by ANOVA and Tukey's test. Different letters in each metabolite row indicate significant differences among the genotypes (*P* < 0.05). These analyses were carried out using the Metaboanalyst platform (*n* = 4). The data from tobacco GCs subjected to Dark or WL are derived from Lima *et al*. (2023).

Aiming to understand how specific the metabolic responses to BL are, we next compared our data with recent results from tobacco GCs exposed to darkness or WL for 0, 10, 20, and 60 min (Lima *et al*., 2023). Data from the 13 metabolites detected across all samples were normalized according to the control (0 min) of each genotype and treatment. Our analysis revealed distinct responses to BL in both cowpea and tobacco GCs compared with tobacco under WL or darkness, as evidenced by the separation of these groups by Component 1 of PLS‐DAs (Fig. 7a–c). Interestingly, the levels of sucrose, glucose, and GABA were lower, while lactate and fumarate were higher in tobacco GCs under WL than BL (Fig. 7d). However, at 20 and 60 min, almost all 13 metabolites showed higher levels under BL than WL in tobacco, with the exception of fumarate (Fig. 7e,f). At 60 min, both cowpea and tobacco GCs showed higher levels of glycine, serine, GABA, and glucose under BL than tobacco GCs under dark or WL conditions. Furthermore, malate and fumarate preferentially accumulated in tobacco GCs under BL and WL, respectively (Fig. 7f). Taken together, the results of this study suggest that the metabolic changes in GCs triggered by light are highly heterogeneous, depending on the species, light quality, and experimental conditions.

## Discussion

### Blue light stomatal responses in cowpea and tobacco

Blue light is a well‐known stomatal opening signal (Zeiger & Hepler, 1977). BL perception by GC plasma membrane‐located phototropins triggers a signalling pathway that modulates metabolic and ionic changes within GCs and ultimately the opening of the stomatal pore (Shimazaki *et al*., 2007). Although BL‐induced stomatal responses have been observed in the basal lineage of plants (Doi *et al*., 2015), either not all plant species respond to it, including certain angiosperms, or certain growth/experimental conditions or certain methods to measure the stomatal opening are not able to identify BL stomatal responses (Matthews *et al*., 2020; Vialet‐Chabrand *et al*., 2021). Despite several controversies found in the literature concerning BL stomatal responses in angiosperms, Arabidopsis stomata are clearly responsive to BL, as demonstrated by several studies (Matthews *et al*., 2020). Although tobacco stomata have been shown to be insensitive to BL in a particular study (Loreto *et al*., 2009), slight increases in stomatal conductance (*g*_s_) were observed when BL was supplemented in leaves previously acclimated to a 90 : 10 proportion of RL and BL (Merilo *et al*., 2014). Furthermore, increases in *g*_s_ were observed in both cowpea and tobacco plants in response to BL (Vialet‐Chabrand *et al*., 2021). Here, we demonstrated using distinct methodologies and plants grown under different conditions that the stomata from both cowpea and tobacco respond to BL, but the increases in *g*_s_ and the speed of stomatal opening induced by BL were higher in tobacco than in cowpea (Fig. S2a,b). However, the kinetics of the stomatal aperture was similar between the species after 60 min under BL (Fig. 1a,b). The differences between the dynamics of stomatal aperture and *g*_s_ may be associated with the intrinsic differences between these analyses and/or the conditions in which the plants were grown, which could have differentially affected the effectiveness of BL perception and/or GC metabolism between these species (Vialet‐Chabrand *et al*., 2021).

### Blue light‐induced guard cell starch remobilization is likely a species‐specific mechanism and highly influenced by the environment/experimental conditions

Guard cell starch metabolism has long been implicated in the regulation of stomatal movements, given that a positive correlation between starch level and stomatal aperture has been observed in certain species (Outlaw & Manchester, 1979; Schnabl, 1980). Additionally, reverse genetic studies indicate that starch synthesis and degradation in GCs are involved in stomatal closure induced by high CO_2_ and stomatal opening induced by BL in Arabidopsis, respectively (Azoulay‐Shemer *et al*., 2016; Horrer *et al*., 2016). However, starch degradation was not observed in GCs of both *V. faba* and *Allium cepa* under BL (Ogawa, 1981). Similarly, our results showed that starch was not degraded in the first 60 min under BL in Arabidopsis, cowpea, and tobacco GCs, while stomatal aperture increased significantly after 60 min under BL (Figs S1a,b, 1a–d). In agreement with our observations, starch degradation was also not observed in tobacco GCs during the dark‐to‐white light transition (Daloso *et al*., 2015). In mesophyll cells, starch is degraded in the range of several minutes to hours, and the speed of starch degradation is highly influenced by the circadian rhythm and the levels of sucrose and trehalose‐6‐phosphate (Martins *et al*., 2013; dos Anjos *et al*., 2018). This suggests that the kinetics of stomatal aperture is faster than starch degradation in illuminated GCs. It seems unlikely, therefore, that starch‐derived metabolites are the major contributors to the initial stomatal opening induced by BL, at least in the species and experimental conditions used here.

It is important to highlight that our starch analysis was carried out using a well‐established enzymatic assay that provides the content of starch in the tissue (Trethewey *et al*., 1998). However, our analysis did not refer to the concentration of starch within GCs. Furthermore, a tendency to reduce GC starch content was observed in tobacco plants during the predawn to the first hours under natural sunlight transition in tobacco (Antunes *et al*., 2017), similar to the results observed in one of the experiments performed here (Fig. S3b). Thus, while our results suggest that BL‐induced starch degradation may not be a common mechanism observed across all angiosperm GCs, it seems that this response is highly influenced by the environment, similar to that observed in mesophyll cells (Annunziata *et al*., 2017). Alternatively, the dynamic nature of GC carbohydrate metabolism, especially considering the source–sink characteristic of GC starch metabolism (Flütsch *et al*., 2022; Piro *et al*., 2023), and a potential rapid starch turnover, with simultaneous starch synthesis and degradation in GCs, may have masked subtle changes in starch content that were not detected by our analysis. In this context, the synthesis of starch has been proposed to occur under stomatal closure conditions (Outlaw, 2003; Santelia & Lawson, 2016). This does not implicate, however, that gluconeogenesis is activated only under such conditions, given that the labelled carbon from ^14^CO_2_, ^14^C‐malate, and H^13^CO_3_ has been identified in starch or glucose under both dark and light conditions (Schnabl, 1980; Lima *et al*., 2021, 2023). This indicates that GC metabolism is highly dynamic and seems to be in a constant nonsteady state condition, which makes it difficult not only to understand the experimental observations but also to detect small changes in the content of the metabolites. Therefore, our results do not implicate that starch metabolism is unimportant for the regulation of Arabidopsis, cowpea, and tobacco GC metabolism during BL‐induced stomatal opening. Starch‐derived metabolites could be responsible for maintaining the stomata opened, after a rapid increase in stomatal opening mediated by the accumulation of K^+^ and its counterions and the degradation of sugars, lipids, and other carbon sources within GCs (Daloso *et al*., 2015; McLachlan *et al*., 2016; Medeiros *et al*., 2018; Lima *et al*., 2023; Auler *et al*., 2024). In fact, sucrose was negatively correlated with stomatal aperture in both Arabidopsis and cowpea (Figs S6, S8), in agreement with the idea that the role of sucrose for stomatal movement regulation, including in response to BL, is primarily energetic.

### Metabolic aspects underpinning blue light‐induced stomatal opening

Guard cell metabolism is highly complex, combining features from source and sink tissues, and is highly sensitive to multiple endogenous and external signals, which create a challenge to understand and model its metabolism since it combines species‐specific responses (Daloso *et al*., 2016a; Flütsch & Santelia, 2021). Although our knowledge of the regulation of GC metabolism has increased recently, studies aiming to investigate the BL‐induced metabolic changes are rare and have recently focussed on starch and lipid metabolisms (McLachlan *et al*., 2016; Dang *et al*., 2024). A previous study showed that the stomatal aperture induced by low BL intensity (10 μmol m^−2^ s^−1^) was positively correlated with the accumulation of sucrose over 120 min. This study also showed that malate levels increased in the first 30 min, then decreased until 120 min in *V. faba* GCs (Talbott & Zeiger, 1993). Neither of these responses were observed here. Additionally, our results indicate that the dynamic of the BL‐induced stomatal opening was associated with different metabolic changes in tobacco and cowpea (Fig. 5). While cowpea metabolites showed a pattern of continuous increase or decrease over time, tobacco metabolites showed a highly heterogeneous dynamic over time (Fig. 2c,d). For instance, the level of all metabolites in the VIP score list increased from 0 to 30 min, decreased from 30 to 40 min, and increased from 40 to 60 min in tobacco (Fig. 2c). These results are in close agreement with previous results, highlighting that tobacco GC metabolism is highly dynamic (Daloso *et al*., 2015, 2016b). Furthermore, our analysis highlights that the changes in nine primary metabolites are substantially different among Arabidopsis, cowpea, and tobacco (Fig. 6a,b). Taken together, our metabolite profiling results indicate that BL‐induced metabolic changes may be species‐specific and/or range according to the experimental condition. Given that the BL signalling pathway is highly conserved among land plants (Doi *et al*., 2015; Westbrook & McAdam, 2020), it seems unlikely that the species‐specific BL metabolic responses are associated with molecular mechanisms such as the presence or absence of BL photoreceptors and/or signalling components among the species studied here. Why these species have differential metabolic responses to BL is unclear, but it could be related to specific biochemical and/or posttranslational mechanisms that regulate starch and primary metabolisms in GCs of these species.

Despite the species‐specific metabolic responses to BL observed here, it is interesting to highlight that sucrose and glucose were positively correlated with metabolites of, or associated with, the TCA cycle in cowpea and tobacco and that sucrose, glucose, and fructose were positively correlated with fumarate and fructose was positively correlated with malate in Arabidopsis (Figs S6–S8). Thus, it seems that the connection between sugars and TCA cycle‐related metabolites, which has several precedents in the literature (Outlaw & Lowry, 1977; Tallman & Zeiger, 1988; Talbott & Zeiger, 1993; Daloso *et al*., 2015), is a common mechanism observed among the species studied here and between WL‐ and BL‐induced stomatal opening. In this context, a previous ^13^C‐labelling study demonstrated that sucrose breakdown is tightly connected to glutamine synthesis in Arabidopsis GCs during the dark‐to‐white light transition (Medeiros *et al*., 2018). Similarly, the levels of both sucrose and glutamate are reduced (Clt4) while pyroglutamate and glucose increased (Clt6‐7) from 20 to 60 min in cowpea GCs under BL (Fig. 4). The activation of the glutamine synthetase (GS)/glutamate synthase (GOGAT) cycle towards glutamine synthesis could be a mechanism to stimulate NO_3_ synthesis, an important K^+^ counterion for GC osmoregulation (Guo *et al*., 2003). Alternatively, higher fluxes towards the GS/GOGAT cycle could be a mechanism to activate fumarase, given that glutamine is an allosteric activator of this enzyme (Zubimendi *et al*., 2018). Additionally, it has been shown that glutamate can induce stomatal closure in Arabidopsis and *V. faba* (Yoshida *et al*., 2016). Thus, the conversion of glutamate into glutamine could be a mechanism to optimize stomatal opening. Indeed, light exposure increased the photosynthetic fluxes towards this pathway in GCs (Lima *et al*., 2023), and this is not observed in leaves (Abadie *et al*., 2017; Daubermann *et al*., 2024). Further studies are needed to unveil the contribution of the GS/GOGAT pathway to the regulation of both GC metabolism and stomatal movements.

## Competing interests

None declared.

## Author contributions

HB, EGM, LA, MEG, WCA and DMD designed the research and experiments. HB, FBSF, EGM, MEG, WCA and VFL performed the experiments. All authors contributed to write the final manuscript. DMD obtained funding. HB and EGM contributed equally to this work.

## Disclaimer

The New Phytologist Foundation remains neutral with regard to jurisdictional claims in maps and in any institutional affiliations.

## Supporting information


**Dataset S1** Metabolic data.

**Fig. S1** Stomatal aperture and starch content in Arabidopsis guard cell‐enriched epidermal fragments subjected to 0 or 60 min under blue light.
**Fig. S2** Stomatal conductance kinetics under blue light in *Nicotiana tabacum* L. (tobacco) and *Vigna unguiculata* L. Walp. (cowpea).
**Fig. S3** Starch content in guard cells of cowpea, tobacco, and Arabidopsis in the dark and after 60 min under blue light.
**Fig. S4** Heat map representation of the changes in metabolite profiling of guard cells harvested after 0 and 60 min under blue light.
**Fig. S5** Heat map representation of the changes in metabolite profiling of tobacco and cowpea guard cells harvested after 0, 10, 20, 30, 40, and 60 min of the dark‐to‐blue light transition.
**Fig. S6** Heat map representation of Pearson's correlation analyses carried out among stomatal aperture parameters and metabolite profiling data from cowpea guard cells.
**Fig. S7** Heat map representation of Pearson's correlation analyses carried out among stomatal aperture parameters and metabolite profiling data from tobacco guard cells.
**Fig. S8** Heat map representation of Pearson's correlation analyses carried out among stomatal aperture and metabolite profiling data from Arabidopsis guard cells subjected to 0 and 60 min under blue light.
Please note: Wiley is not responsible for the content or functionality of any Supporting Information supplied by the authors. Any queries (other than missing material) should be directed to the *New Phytologist* Central Office.

## Data Availability

All relevant data can be found within the manuscript. The metabolomics data can be found in Dataset S1.

## References

[nph70257-bib-0001] Abadie C , Lothier J , Boex‐Fontvieille E , Carroll A , Tcherkez G . 2017. Direct assessment of the metabolic origin of carbon atoms in glutamate from illuminated leaves using 13C‐NMR. New Phytologist 216: 1079–1089. 28771732 10.1111/nph.14719

[nph70257-bib-0002] Allaway WG . 1973. Accumulation of malate in guard cells of *Vicia faba* during stomatal opening. Planta 70: 63–70. 10.1007/BF00386923 24474312

[nph70257-bib-0003] Ando E , Kinoshita T . 2018. Red light‐induced phosphorylation of plasma membrane H^+^ ‐ATPase in stomatal guard cells. Plant Physiology 178: 838–849. 30104254 10.1104/pp.18.00544 PMC6181031

[nph70257-bib-0004] dos Anjos L , Pandey PK , Moraes TA , Feil R , Lunn JE , Stitt M . 2018. Feedback regulation by trehalose 6‐phosphate slows down starch mobilization below the rate that would exhaust starch reserves at dawn in Arabidopsis leaves. Plant Direct 2: e00078. 31245743 10.1002/pld3.78 PMC6508811

[nph70257-bib-0005] Annunziata MG , Apelt F , Carillo P , Krause U , Feil R , Mengin V , Lauxmann MA , Köhl K , Nikoloski Z , Stitt M *et al*. 2017. Getting back to nature: a reality check for experiments in controlled environments. Journal of Experimental Botany 68: 4463–4477. 28673035 10.1093/jxb/erx220 PMC5853417

[nph70257-bib-0006] Antunes WC , de Menezes Daloso D , Pinheiro DP , Williams TCR , Loureiro ME . 2017. Guard cell‐specific down‐regulation of the sucrose transporter SUT1 leads to improved water use efficiency and reveals the interplay between carbohydrate metabolism and K+ accumulation in the regulation of stomatal opening. Environmental and Experimental Botany 135: 73–85.

[nph70257-bib-0007] Auler PA , Lemos M d S , Porto NP , Mendes K d R , Bret RSC , Daloso DM . 2024. Abscisic acid‐mediated guard cell metabolism regulation. Plant Physiology and Biochemistry 214: 108889. 38954945 10.1016/j.plaphy.2024.108889

[nph70257-bib-0008] Azoulay‐Shemer T , Bagheri A , Wang C , Palomares A , Stephan AB , Kunz HH , Schroeder JI . 2016. Starch biosynthesis in guard cells but not in mesophyll cells is involved in CO_2_‐induced stomatal closing. Plant Physiology 171: 788–798. 27208296 10.1104/pp.15.01662 PMC4902578

[nph70257-bib-0009] Cândido‐Sobrinho SA , Lima VF , Freire FBS , de Souza LP , Gago J , Fernie AR , Daloso DM . 2022. Metabolism‐mediated mechanisms underpin the differential stomatal speediness regulation among ferns and angiosperms. Plant, Cell & Environment 45: 296–311. 10.1111/pce.14232 34800300

[nph70257-bib-0010] Daloso DM , Antunes WC , Pinheiro DP , Waquim JP , Araújo WL , Loureiro ME , Fernie AR , Williams TCR . 2015. Tobacco guard cells fix CO_2_ by both Rubisco and PEPcase while sucrose acts as a substrate during light‐induced stomatal opening. Plant, Cell & Environment 38: 2353–2371. 10.1111/pce.12555 25871738

[nph70257-bib-0011] Daloso DM , dos Anjos L , Fernie AR . 2016a. Roles of sucrose in guard cell regulation. New Phytologist 211: 809–818. 27060199 10.1111/nph.13950

[nph70257-bib-0012] Daloso DM , Medeiros DB , dos Anjos L , Yoshida T , Araújo WL , Fernie AR . 2017. Metabolism within the specialized guard cells of plants. New Phytologist 216: 1018–1033. 28984366 10.1111/nph.14823

[nph70257-bib-0013] Daloso DM , Williams TCR , Antunes WC , Pinheiro DP , Müller C , Loureiro ME , Fernie AR . 2016b. Guard cell‐specific upregulation of sucrose synthase 3 reveals that the role of sucrose in stomatal function is primarily energetic. New Phytologist 209: 1470–1483. 26467445 10.1111/nph.13704

[nph70257-bib-0014] Dang T , Piro L , Pasini C , Santelia D . 2024. Starch metabolism in guard cells: at the intersection of environmental stimuli and stomatal movement. Plant Physiology 196: 1758–1777. 39115378 10.1093/plphys/kiae414 PMC11531838

[nph70257-bib-0015] Daubermann AG , Lima VF , Erban A , Kopka J , Fernie AR , Schwarzländer M , dos Anjos L , Daloso DM . 2024. Novel guard cell sink characteristics revealed by a multi‐species/cell‐types meta‐analysis of ^13^C‐labelling experiments. Theoretical and Experimental Plant Physiology 36: 1–20.

[nph70257-bib-0016] Ding M , Zhang M , Zeng H , Hayashi Y , Zhu Y , Kinoshita T . 2021. Molecular basis of plasma membrane H+‐ATPase function and potential application in the agricultural production. Plant Physiology and Biochemistry 168: 10–16. 34607207 10.1016/j.plaphy.2021.09.036

[nph70257-bib-0017] Dittrich P , Raschke K . 1977. Malate metabolism in isolated epidermis of *Commelina communis* L. in relation to stomatal functioning. Planta 134: 77–81. 24419583 10.1007/BF00390098

[nph70257-bib-0018] Doi M , Kitagawa Y , Shimazaki K . 2015. Stomatal blue light response is present in early vascular plants. Plant Physiology 169: 1205–1213. 26307440 10.1104/pp.15.00134 PMC4587438

[nph70257-bib-0019] Evans JR , Lawson T . 2020. From green to gold: agricultural revolution for food security. Journal of Experimental Botany 71: 2211–2215. 32251509 10.1093/jxb/eraa110

[nph70257-bib-0020] Falcioni R , Moriwaki T , Perez‐Llorca M , Munné‐Bosch S , Gibin MS , Sato F , Pelozo A , Pattaro MC , Giacomelli ME , Rüggeberg M *et al*. 2020. Cell wall structure and composition is affected by light quality in tomato seedlings. Journal of Photochemistry and Photobiology B: Biology 203: 111745. 31931381 10.1016/j.jphotobiol.2019.111745

[nph70257-bib-0021] Fischer R . 1968. Stomatal opening: role of potassium uptake by guard cells. Science 160: 784–785. 5646418 10.1126/science.160.3829.784

[nph70257-bib-0022] Flütsch S , Horrer D , Santelia D . 2022. Starch biosynthesis in guard cells has features of both autotrophic and heterotrophic tissues. Plant Physiology 189: 541–556. 35238373 10.1093/plphys/kiac087 PMC9157084

[nph70257-bib-0023] Flütsch S , Nigro A , Conci F , Fajkus J , Thalmann M , Trtílek M , Panzarová K , Santelia D . 2020. Glucose uptake to guard cells via STP transporters provides carbon sources for stomatal opening and plant growth. EMBO Reports 21: 1–13. 10.15252/embr.201949719 PMC7403697 32627357

[nph70257-bib-0024] Flütsch S , Santelia D . 2021. Mesophyll‐derived sugars are positive regulators of light‐driven stomatal opening. New Phytologist 230: 1754–1760. 33666260 10.1111/nph.17322

[nph70257-bib-0025] Guo F‐Q , Young J , Crawford NM . 2003. The nitrate transporter AtNRT1.1 (CHL1) functions in stomatal opening and contributes to drought susceptibility in Arabidopsis. Plant Cell 15: 107–117. 12509525 10.1105/tpc.006312 PMC143464

[nph70257-bib-0026] Heath OVS . 1947. Role of starch inlight‐indiced stomatal movement, and a new reagent for staining stomatal starch. Nature 159: 647–648. 20239728 10.1038/159647b0

[nph70257-bib-0027] Hoagland DR , Arnon DI . 1950. The water‐culture method for growing plants without soil. Berkeley, CA, USA: The College of Agriculture.

[nph70257-bib-0028] Horrer D , Flu S , Leonhardt N , Santelia D , Horrer D , Flu S . 2016. Blue light induces a distinct starch degradation pathway in guard cells for stomatal opening. Current Biology 26: 362–370. 26774787 10.1016/j.cub.2015.12.036

[nph70257-bib-0029] Kopka J , Schauer N , Krueger S , Birkemeyer C , Usadel B , Bergmüller E , Dörmann P , Weckwerth W , Gibon Y , Stitt M *et al*. 2005. GMD@CSB.DB: the Golm metabolome database. Bioinformatics 21: 1635–1638. 15613389 10.1093/bioinformatics/bti236

[nph70257-bib-0030] Lasceve G , Leymarie J , Vavasseur A . 1997. Alterations in light‐induced stomatal opening in the starch‐deficient mutant of *Arabidopsis thaliana* L. deficient in chloroplasts phophoglucomutase activity. Plant, Cell & Environment 20: 350–358.

[nph70257-bib-0031] Lima VF , dos Anjos L , Medeiros DB , Cândido‐Sobrinho SA , Souza LP , Gago J , Fernie AR , Daloso DM . 2019. The sucrose‐to‐malate ratio correlates with the faster CO_2_ and light stomatal responses of angiosperms compared to ferns. New Phytologist 223: 1873–1887. 31099898 10.1111/nph.15927

[nph70257-bib-0032] Lima VF , Erban A , Daubermann AG , Freire FBS , Porto NP , Cândido‐Sobrinho SA , Medeiros DB , Schwarzländer M , Fernie AR , Anjos L *et al*. 2021. Establishment of a GC‐MS‐based ^1^3C‐positional isotopomer approach suitable for investigating metabolic fluxes in plant primary metabolism. The Plant Journal 108: 1213–1233. 34486764 10.1111/tpj.15484

[nph70257-bib-0033] Lima VF , Freire FBS , Cândido‐Sobrinho SA , Porto NP , Medeiros DB , Erban A , Kopka J , Schwarzländer M , Fernie AR , Daloso DM . 2023. Unveiling the dark side of guard cell metabolism. Plant Physiology and Biochemistry 201: 107862. 37413941 10.1016/j.plaphy.2023.107862

[nph70257-bib-0034] Lima VF , Medeiros DB , Dos Anjos L , Gago J , Fernie AR , Daloso DM . 2018. Toward multifaceted roles of sucrose in the regulation of stomatal movement. Plant Signaling & Behavior 13: e1494468. 30067434 10.1080/15592324.2018.1494468 PMC6149408

[nph70257-bib-0035] Lisec J , Schauer N , Kopka J , Willmitzer L , Fernie AR . 2006. Gas chromatography mass spectrometry‐based metabolite profiling in plants. Nature Protocols 1: 387–396. 17406261 10.1038/nprot.2006.59

[nph70257-bib-0036] Lloyd FE . 1908. The physiology of stomata, vol. 82. Washington, DC, USA: Publications of the Carnegie Institution of Washington, 1–42.

[nph70257-bib-0037] Loreto F , Tsonev T , Centritto M . 2009. The impact of blue light on leaf mesophyll conductance. Journal of Experimental Botany 60: 2283–2290. 19395388 10.1093/jxb/erp112

[nph70257-bib-0038] Martins MCM , Hejazi M , Fettke J , Steup M , Feil R , Krause U , Arrivault S , Vosloh D , Figueroa CM , Ivakov A *et al*. 2013. Feedback inhibition of starch degradation in Arabidopsis leaves mediated by trehalose 6‐phosphate. Plant Physiology 163: 1142–1163. 24043444 10.1104/pp.113.226787 PMC3813640

[nph70257-bib-0039] Matthews JSA , Vialet‐Chabrand S , Lawson T . 2020. Role of blue and red light in stomatal dynamic behaviour. Journal of Experimental Botany 71: 2253–2269. 31872212 10.1093/jxb/erz563 PMC7134916

[nph70257-bib-0040] McLachlan DH , Lan J , Geilfus CM , Dodd AN , Larson T , Baker A , Hõrak H , Kollist H , He Z , Graham I *et al*. 2016. The breakdown of stored triacylglycerols is required during light‐induced stomatal opening. Current Biology 26: 707–712. 26898465 10.1016/j.cub.2016.01.019 PMC4791430

[nph70257-bib-0041] Medeiros DB , Perez Souza L , Antunes WC , Araújo WL , Daloso DM , Fernie AR . 2018. Sucrose breakdown within guard cells provides substrates for glycolysis and glutamine biosynthesis during light‐induced stomatal opening. The Plant Journal 94: 583–594. 29543357 10.1111/tpj.13889

[nph70257-bib-0042] Merilo E , Jõesaar I , Brosché M , Kollist H . 2014. To open or to close: species‐specific stomatal responses to simultaneously applied opposing environmental factors. New Phytologist 202: 499–508. 24392838 10.1111/nph.12667

[nph70257-bib-0043] Mott KA . 2009. Opinion: stomatal responses to light and CO_2_ depend on the mesophyll. Plant, Cell & Environment 32: 1479–1486. 10.1111/j.1365-3040.2009.02022.x 19627565

[nph70257-bib-0044] Mott KA , Sibbernsen ED , Shope JC . 2008. The role of the mesophyll in stomatal responses to light and CO_2_ . Plant, Cell and Environment 31: 1299–1306. 10.1111/j.1365-3040.2008.01845.x 18541006

[nph70257-bib-0045] Ogawa T . 1981. Blue light response of stomata with starch‐containing (*Vicia faba*) and starch‐deficient (*Allium cepa*) guard cells under background illumination with red light. Plant Science Letters 22: 103–108.

[nph70257-bib-0046] Ogawa T , Ishikawa H , Shimada K , Shibata K . 1978. Synergistic action of red and blue light and action spectra for malate formation in guard cells of *Vicia faba* L. Planta 142: 61–65. 24407999 10.1007/BF00385121

[nph70257-bib-0047] Outlaw WH , Lowry OH . 1977. Organic acid and potassium accumulation in guard cells during stomatal opening. Proceedings of the National Academy of Sciences, USA 74: 4434–4438. 10.1073/pnas.74.10.4434 PMC431957 16592449

[nph70257-bib-0048] Outlaw WH , Manchester J . 1979. Guard cell starch concentration quantitatively related to stomatal aperture. Plant Physiology 64: 79–82. 16660919 10.1104/pp.64.1.79 PMC543028

[nph70257-bib-0049] Outlaw WHJ . 2003. Integration of cellular and physiological functions of guard cells integration of cellular and physiological functions of guard cells. Critical Reviews in Plant Sciences 22: 503–5229.

[nph70257-bib-0050] Pang Z , Chong J , Zhou G , De Lima Morais DA , Chang L , Barrette M , Gauthier C , Jacques PÉ , Li S , Xia J . 2021. MetaboAnalyst 5.0: narrowing the gap between raw spectra and functional insights. Nucleic Acids Research 49: W388–W396. 34019663 10.1093/nar/gkab382 PMC8265181

[nph70257-bib-0051] Pattaro MC , Falcioni R , Moriwaki T , Corrêa Alves D , Antunes WC . 2024. Blue light strongly promotes de‐etiolation over green, moderate over red, but have limited action over far‐red lights in lettuce plants. Scientia Horticulturae 328: 112863.

[nph70257-bib-0052] Penfield S , Clements S , Bailey KJ , Gilday AD , Leegood RC , Gray JE , Graham IA . 2012. Expression and manipulation of PHOSPHOENOLPYRUVATE CARBOXYKINASE 1 identifies a role for malate metabolism in stomatal closure. The Plant Journal 69: 679–688. 22007864 10.1111/j.1365-313X.2011.04822.x

[nph70257-bib-0053] Piro L , Flütsch S , Santelia D . 2023. Arabidopsis Sucrose Synthase 3 (SUS3) regulates starch accumulation in guard cells at the end of day. Plant Signaling & Behavior 18: 4–8. 10.1080/15592324.2023.2171614 PMC9928453 36774587

[nph70257-bib-0054] Santelia D , Lawson T . 2016. Rethinking guard cell metabolism. Plant Physiology 172: 1371–1392. 27609861 10.1104/pp.16.00767 PMC5100799

[nph70257-bib-0055] Schnabl H . 1980. CO_2_ and malate metabolism in starch‐containing and starch‐lacking guard‐cell protoplasts. Planta 149: 52–58. 24306192 10.1007/BF00386227

[nph70257-bib-0056] Shimazaki K , Doi M , Assmann SM , Kinoshita T . 2007. Light regulation of stomatal movement. Annual Review of Plant Biology 58: 219–247. 10.1146/annurev.arplant.57.032905.105434 17209798

[nph70257-bib-0057] Sussmilch FC , Schultz J , Hedrich R , Roelfsema MRG . 2019. Acquiring control: the evolution of stomatal signalling pathways. Trends in Plant Science 24: 342–351. 30797685 10.1016/j.tplants.2019.01.002

[nph70257-bib-0058] Szecowka M , Heise R , Tohge T , Nunes‐Nesi A , Vosloh D , Huege J , Feil R , Lunn J , Nikoloski Z , Stitt M *et al*. 2013. Metabolic fluxes in an illuminated Arabidopsis rosette. Plant Cell 25: 694–714. 23444331 10.1105/tpc.112.106989 PMC3608787

[nph70257-bib-0059] Talbott LD , Zeiger E . 1993. Sugar and organic acid accumulation in guard cells of *Vicia faba* in response to red and blue light. Plant Physiology 102: 1163–1169. 12231893 10.1104/pp.102.4.1163 PMC158901

[nph70257-bib-0060] Tallman G , Zeiger E . 1988. Light quality and osmoregulation in vicia guard cells: evidence for involvement of three metabolic pathways. Plant Physiology 88: 887–895. 16666400 10.1104/pp.88.3.887 PMC1055678

[nph70257-bib-0061] Trethewey RN , Geigenberger P , Riedel K , Hajirezaei MR , Sonnewald U , Stitt M , Riesmeier JW , Willmitzer L . 1998. Combined expression of glucokinase and invertase in potato tubers leads to a dramatic reduction in starch accumulation and a stimulation of glycolysis. The Plant Journal 15: 109–118. 19422146 10.1046/j.1365-313x.1998.00190.x

[nph70257-bib-0062] Van Kirk CA , Raschke K . 1978. Release of malate from epidermal strips during stomatal closure. Plant Physiology 61: 474–475. 16660318 10.1104/pp.61.3.474 PMC1091893

[nph70257-bib-0063] Vialet‐Chabrand S , Matthews JSA , Lawson T . 2021. Light, power, action! Interaction of respiratory energy‐ and blue light‐induced stomatal movements. New Phytologist 231: 2231–2246. 34101837 10.1111/nph.17538

[nph70257-bib-0064] Von Mohl H . 1856. Welche Ursachen bewirken die Erweiterung und Verengung der Spaltoffnungen? Botanische Zeitung 14: 697–704.

[nph70257-bib-0065] Westbrook AS , McAdam SAM . 2020. Atavistic stomatal responses to blue light in Marsileaceae. Plant Physiology 184: 1378–1388. 32843522 10.1104/pp.20.00967 PMC7608159

[nph70257-bib-0066] Xia J , Wishart DS . 2011. Web‐based inference of biological patterns, functions and pathways from metabolomic data using MetaboAnalyst. Nature Protocols 6: 743–760. 21637195 10.1038/nprot.2011.319

[nph70257-bib-0067] Yoshida R , Mori IC , Kamizono N , Shichiri Y , Shimatani T , Miyata F , Honda K , Iwai S . 2016. Glutamate functions in stomatal closure in Arabidopsis and fava bean. Journal of Plant Research 129: 39–49. 26586261 10.1007/s10265-015-0757-0 PMC5515988

[nph70257-bib-0068] Zeiger E , Hepler PK . 1977. Light and stomatal function: blue light stimulates swelling of guard cell protoplasts. Science 196: 887–889. 17821809 10.1126/science.196.4292.887

[nph70257-bib-0069] Zubimendi JP , Martinatto A , Valacco MP , Moreno S , Andreo CS , Drincovich MF , Tronconi MA . 2018. The complex allosteric and redox regulation of the fumarate hydratase and malate dehydratase reactions of *Arabidopsis thaliana* Fumarase 1 and 2 gives clues for understanding the massive accumulation of fumarate. FEBS Journal 285: 2205–2224. 29688630 10.1111/febs.14483

