# Supplementary material for: Blue light‐induced stomatal opening is associated with species‐specific changes in primary metabolism but not with starch breakdown in guard cells
Source: New Phytol. 2025 Jun 11;248(5):2347–60. doi: 10.1111/nph.70257 (PMC12589703; doi:10.1111/nph.70257)
Supplement: Supplementary file 2 — Fig. S1 Stomatal aperture and starch content in Arabidopsis guard cell‐enriched epidermal fragments subjected to 0 or 60 min under blue light. Fig. S2 Stomatal conductance kinetics under blue light in Nicotiana tabacum L. (tobacco) and Vigna unguiculata L. Walp. (cowpea). Fig. S3 Starch content in guard cells of cowpea, tobacco, and Arabidopsis in the dark and after 60 min under blue light. Fig. S4 Heat map representation of the changes in metabolite profiling of guard cells harvested after 0 and 60 min under blue light. Fig. S5 Heat map representation of the changes in metabolite profiling of tobacco and cowpea guard cells harvested after 0, 10, 20, 30, 40, and 60 min of the dark‐to‐blue light transition. Fig. S6 Heat map representation of Pearson's correlation analyses carried out among stomatal aperture parameters and metabolite profiling data from cowpea guard cells. Fig. S7 Heat map representation of Pearson's correlation analyses carried out among stomatal aperture parameters and metabolite profiling data from tobacco guard cells. Fig. S8 Heat map representation of Pearson's correlation analyses carried out among stomatal aperture and metabolite profiling data from Arabidopsis guard cells subjected to 0 and 60 min under blue light. Please note: Wiley is not responsible for the content or functionality of any Supporting Information supplied by the authors. Any queries (other than missing material) should be directed to the New Phytologist Central Office. [file NPH-248-2347-s001.docx]

**New Phytologist Supporting Information Figs S1–S8**

**Article title:** Blue light-induced stomatal opening is associated to species-specific changes in primary metabolism but not to starch breakdown in guard cells

**Authors:** Humaira Bahadar, Eva Gomes Morais, Francisco Bruno S. Freire, [Valéria F. Lima](https://www.researchgate.net/profile/Valeria-Lima-10?utm_content=businessCard&utm_source=profileContributions&rgutm_meta1=AC%3A33151110&_tp=eyJjb250ZXh0Ijp7ImZpcnN0UGFnZSI6ImhvbWUiLCJwYWdlIjoicHJvZmlsZSJ9fQ), Marina Ellen Giacomelli, Leticia dos Anjos, Werner Camargos Antunes, Danilo M. Daloso.

**Article acceptance date:** 6 May 2025

**The following Supporting Information is available for this article:**

**Figure S1**.  Stomatal aperture and starch content in Arabidopsis guard cell enriched epidermal fragments subjected to 0 or 60 min under blue light. A pool of guard cell-enriched epidermal fragments was harvested at pre-dawn, stored in a hypertonic solution, and then transferred to blue light (75-90 µmol photons m^-2^ s^-1^). A set of guard cell enriched epidermal fragments was used to analyse the stomatal aperture and frozen for starch analysis immediately after the start of the experiment, which corresponds the time 0 min. After 60 minutes under blue light, another set of guard cell enriched epidermal fragments were harvested in a nylon membrane and used to measure stomatal aperture using a light microscope and frozen for starch analysis. Stomatal aperture data refers to the width of the stomatal pore (µm). Asterisk (*) indicates significant difference between 0 and 60 min under blue light Student´s *t* test (*P* < 0.05). Bars represent average ± standard error (n=4).

**Figure S2** Stomatal conductance (*g*_s_) kinetics under blue light in *Nicotiana tabacum* L. (tobacco) and *Vigna unguiculata* L*. Walp*. (cowpea). **a**) Kinetics of *g*_s_ (mol H_2_O m^-2^ s^-1^) through the dark to blue light transition in tobacco and cowpea. Black and yellow bars above the graph indicates dark and illuminated periods, respectively. **b**) Stomatal opening speediness (mol H_2_O m^-2^ s^-1^ min^-1^) measured as the maximum slope (*Sl*_max_) of the linear phase of the *g*_s_ increase during the dark to blue light transition. Asterisk (*) indicates significant difference between tobacco and cowpea by Student’s *t* test at 5% of probability (*P* < 0.05) (n = 5 ± SE).

**Figure S3** Starch content (µmol g^-1^ FW) in guard cells of cowpea (a), tobacco (b) and Arabidopsis (c) in the dark (0 min) and after 60 min under blue light. Guard cell enriched epidermal fragments were harvested at pre-dawn and immediately submitted to blue light for 0 and 60 minutes. After these time points, the guard cell enriched epidermal fragments were collected in a nylon membrane and frozen in liquid nitrogen. No statistical difference (*P* < 0.05) between 0 and 60 min was observed by Student´s *t* test within the species. Bars represent average ± standard error (n=4).

**Figure S4**. Changes in guard cell primary metabolites of Arabidopsis induced by blue light. a) Heat map representation of the changes in metabolite profiling of guard cells harvested after 0 and 60 min under blue light. Metabolite profiling data was normalized by the average of values found at the time 0 min followed by log2 transformation for heat map representation. Asterisks (*) indicate significant differences between 0 and 60 min by Student’s *t*-test (*P* < 0.05). b) a-b) Partial least square-discriminant analysis (PLS-DA) using the metabolite profiling data displayed in the heatmap. The percentage variation explained by the components 1 and 2 of the PLS-DA is represented in each axis. PLS-DA was carried out using the Metaboanalyst platform (n = 4).

**Figure S5**. Heat map representation of the changes in metabolite profiling of tobacco (a) and cowpea (b) guard cells harvested after 0, 10, 20, 30, 40 and 60 min of the dark-to-blue light transition. Metabolite profiling data were normalized by the average of values found at the time 0 min followed by log2 transformation for heat map representation. Asterisks (*) indicate significant differences from the control (time 0 min) by Student’s *t*-test (*P* < 0.05) (n = 3 to 4).

**Figure S6**. Heat map representation of Pearson correlation analyses carried out among stomatal aperture parameters and metabolite profiling data from cowpea guard cells. Blue and red colours indicate negative and positive correlations, respectively. Asterisks (*) indicate significant correlation (*P* < 0.05). The heat map was generated using the Metaboanalyst platform. Avg W; average stomatal width, Avg L; average stomatal length, Avg WXL; stomatal average width x stomatal average length.

**Figure S7**. Heat map representation of Pearson correlation analyses carried out among stomatal aperture parameters and metabolite profiling data from tobacco guard cells. Blue and red colours indicate negative and positive correlations, respectively. Asterisks (*) indicate significant correlation (*P* < 0.05). The heat map was generated using the Metaboanalyst platform. Avg W; average stomatal width, Avg L; average stomatal length, Avg WXL; stomatal average width x stomatal average length.

**Figure S8**. Heat map representation of Pearson correlation analyses carried out among stomatal aperture and metabolite profiling data from Arabidopsis guard cells subjected to 0 and 60 min under blue light. Blue and red colours indicate negative and positive correlations, respectively. Asterisks (*) indicate significant correlation (*P* < 0.05). The heat map was generated using the Metaboanalyst platform.

**Figure S1**


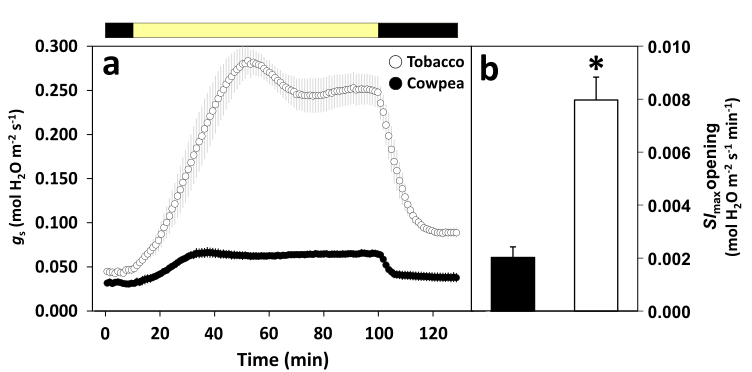


**Figure S2**

**Figure S3**

**
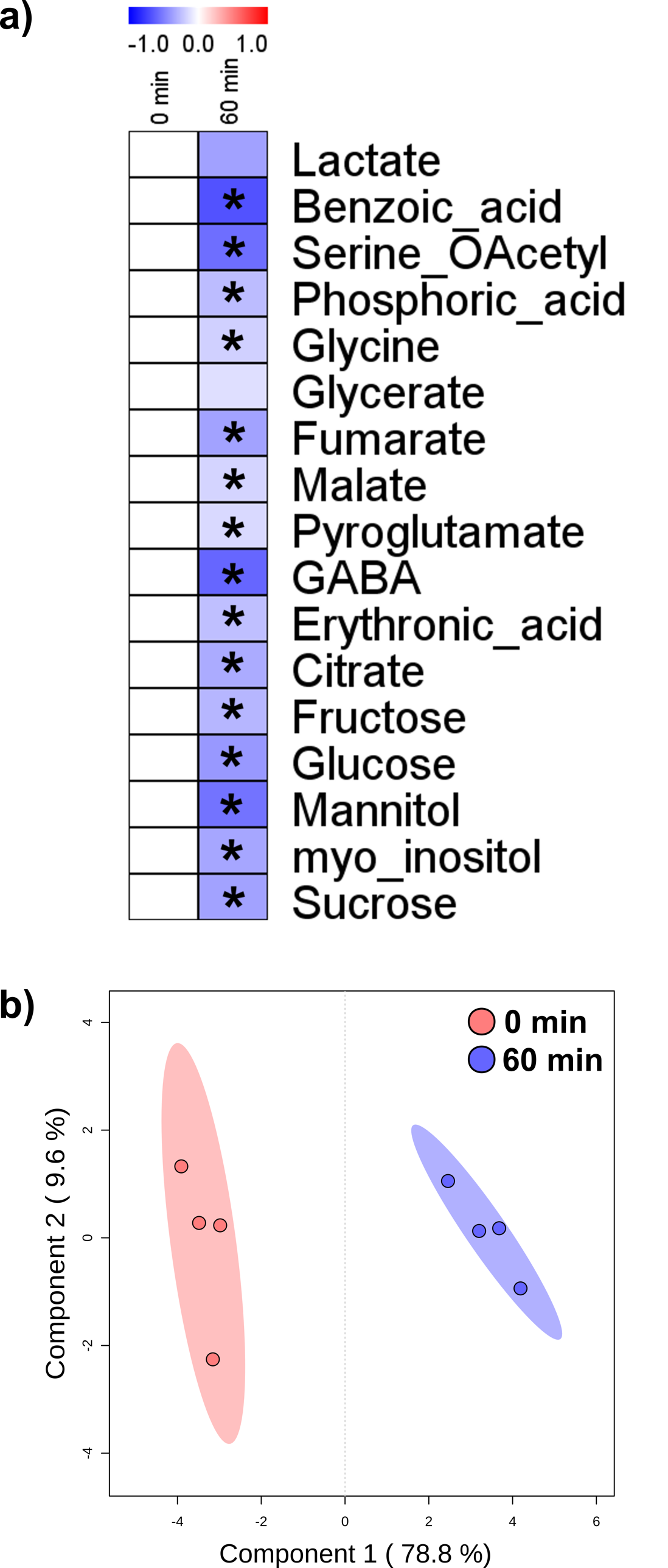
**

**Figure S4**

**
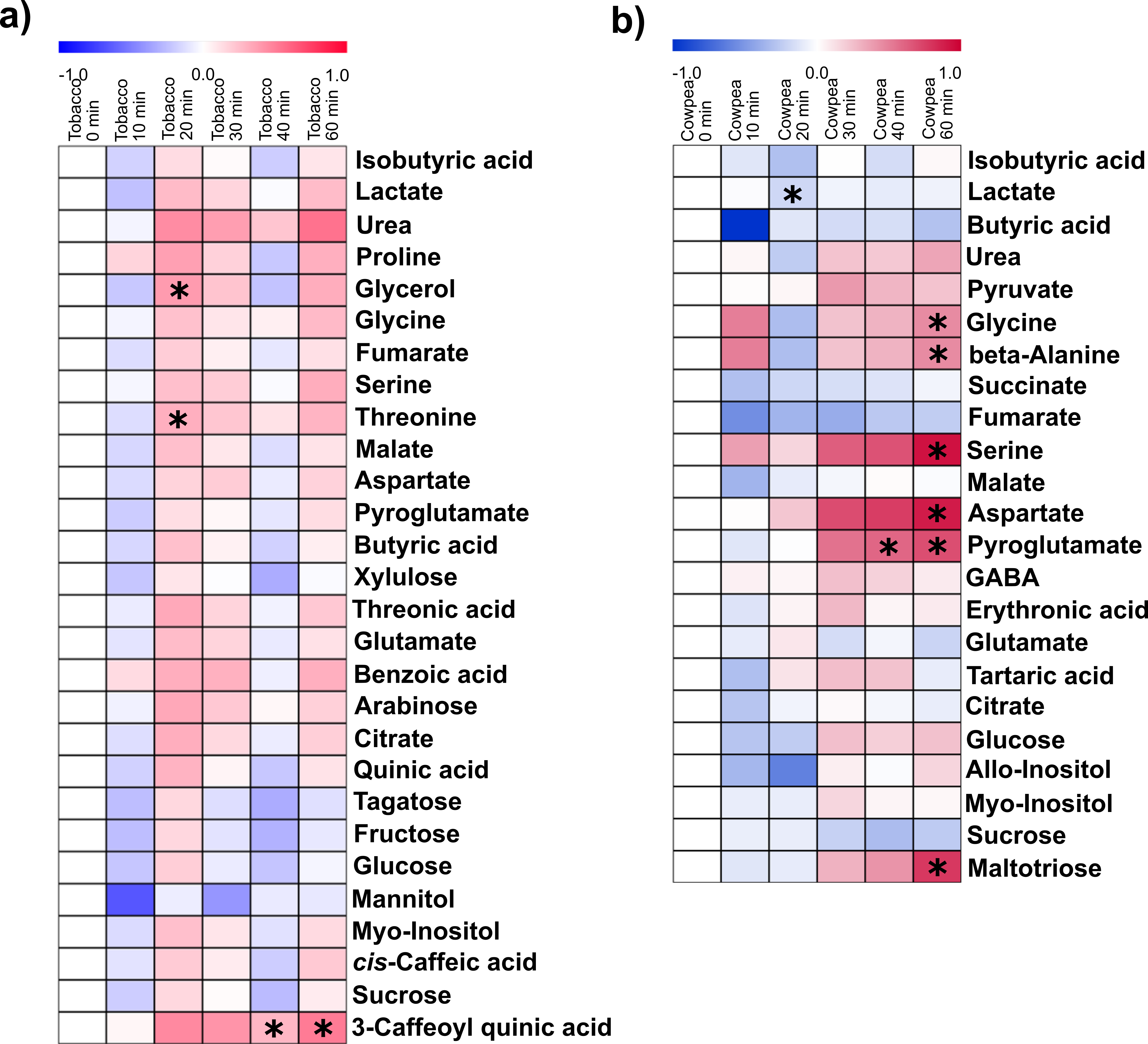
**

**Figure S5**

**
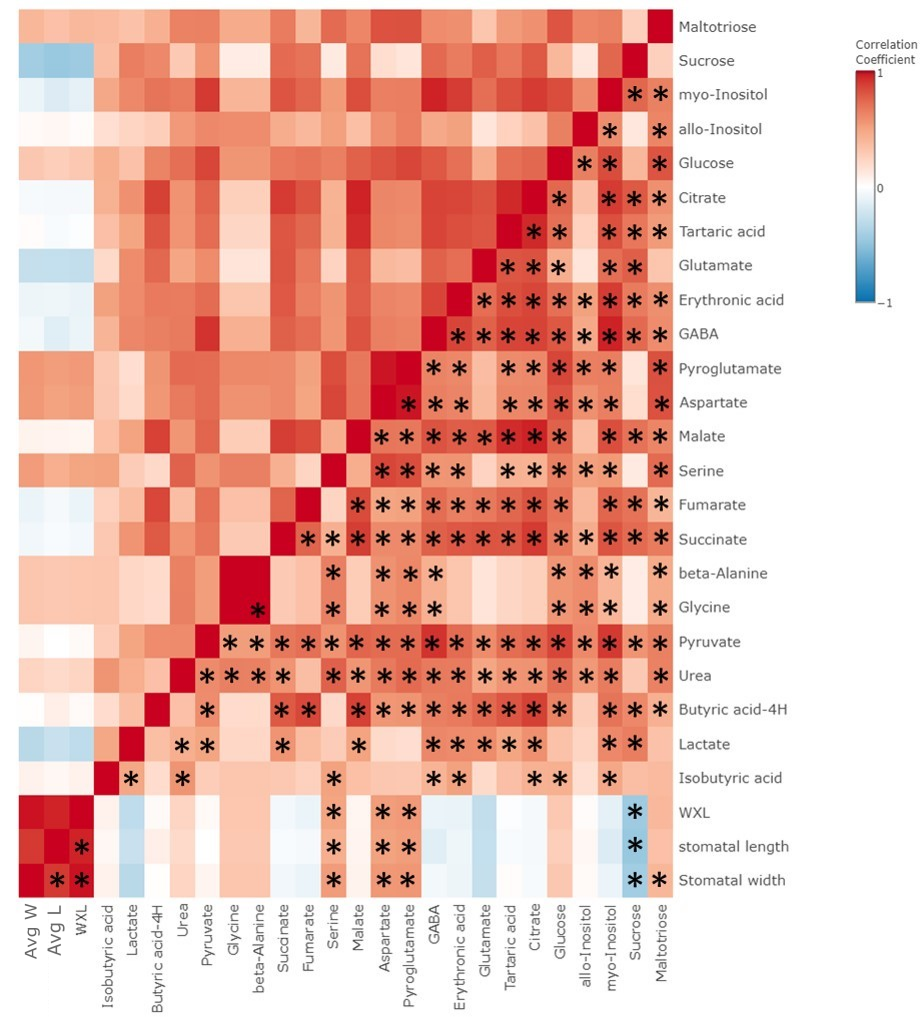
**

**Figure S6**

**
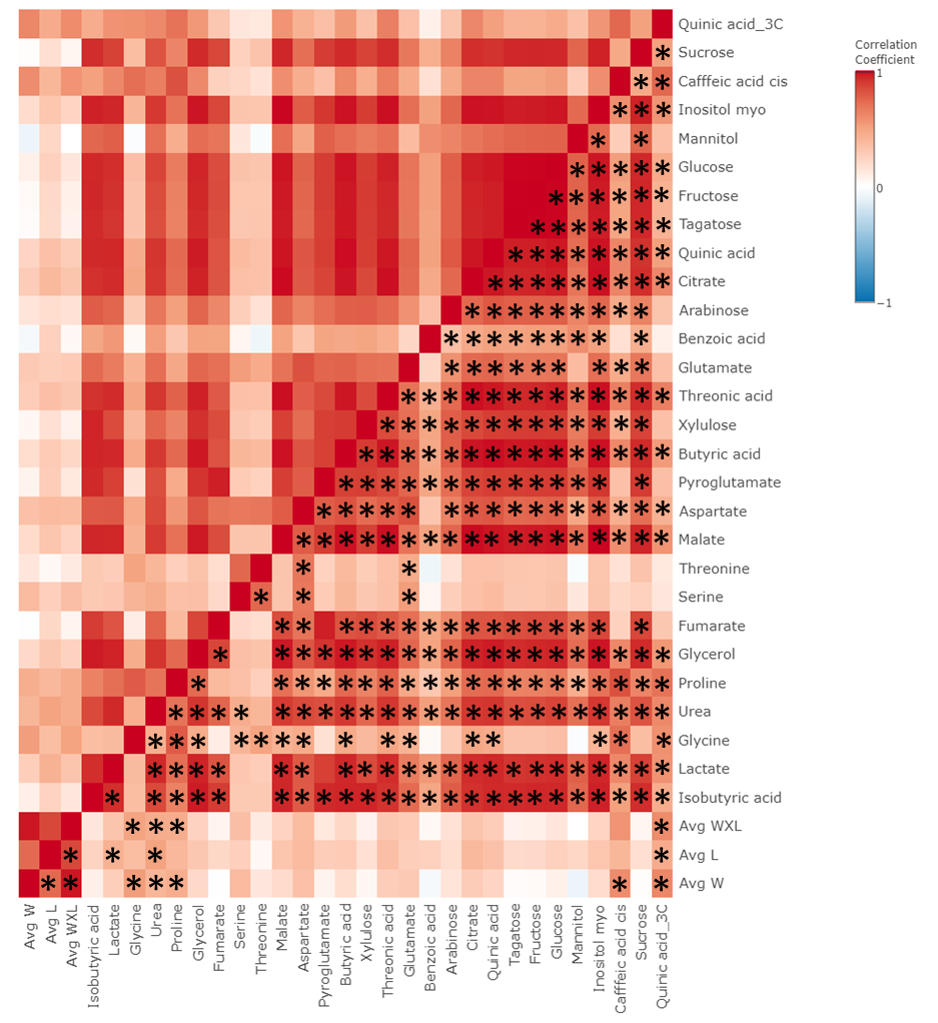
**

**Figure S7**

**
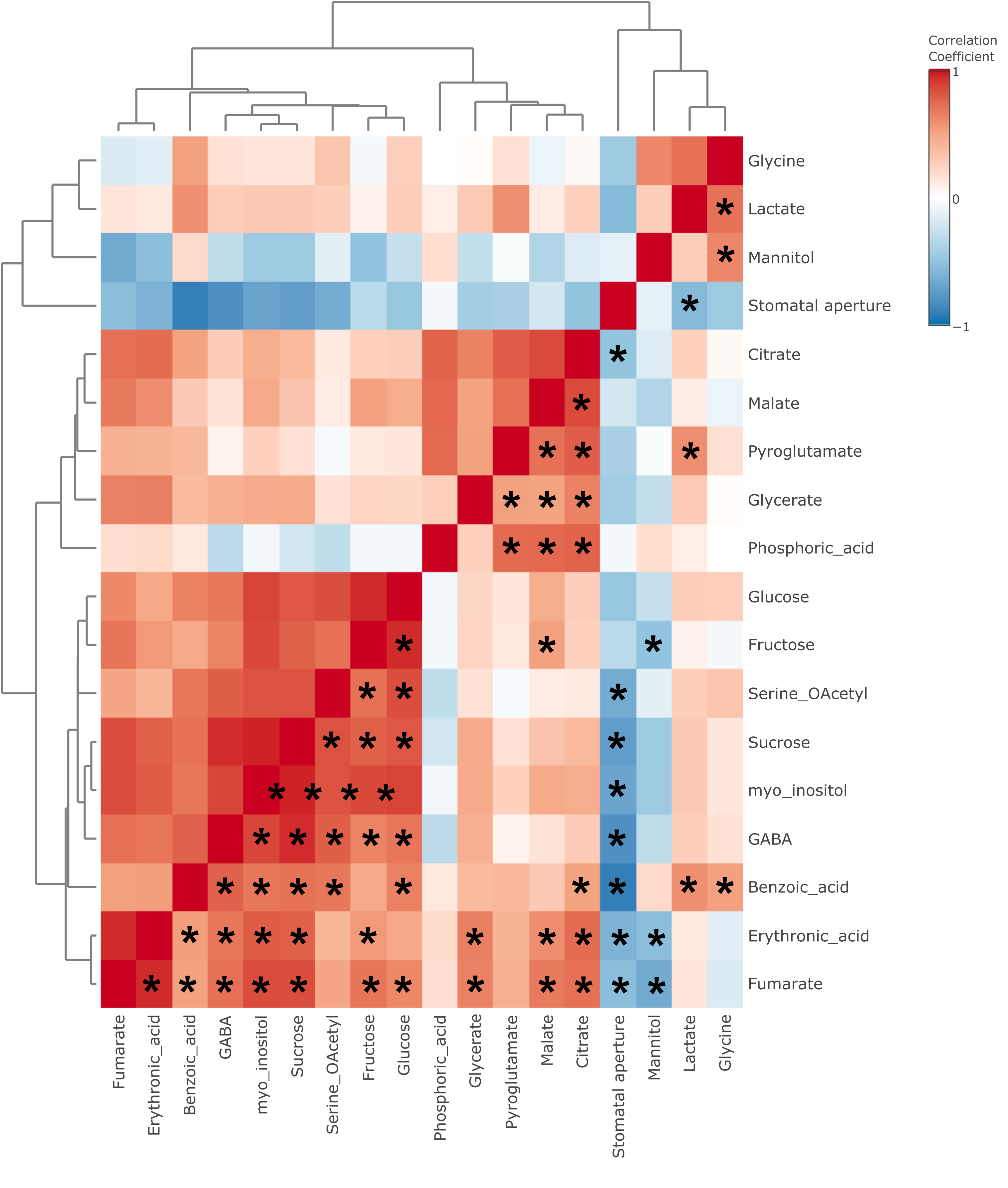
**

**Figure S8**
